# Engineering delivery platforms for CRISPR-Cas and their applications in healthcare, agriculture and beyond

**DOI:** 10.1039/d5na00535c

**Published:** 2026-01-05

**Authors:** Nisha Bharti, Unnati Modi, Dhiraj Bhatia, Raghu Solanki

**Affiliations:** a School of Life Sciences, Central University of Gujarat Kundhela, Taluka-Dabhoi Vadodara Gujarat-391107 India; b Department of Biological Sciences and Engineering, Indian Institute of Technology Gandhinagar Palaj Gandhinagar Gujarat-382355 India raghu.solanki@iitgn.ac.in dhiraj.bhatia@iitgn.ac.in; c School of Biotechnology & Bioengineering, Institute of Advanced Research, Koba Institutional Area Near GIFT City Bridge Gandhinagar Gujarat-382426 India

## Abstract

Clustered regularly interspaced short palindromic repeats (CRISPR)-Cas systems have transformed genome editing through unprecedented precision, and next-generation variants (base and prime editors) further enhance specificity by enabling targeted nucleotide changes without introducing double-strand DNA breaks. These technologies have unlocked broad applications in therapeutic gene correction, functional genomics, infectious disease management, diagnostics, agricultural engineering, environmental biotechnology, and synthetic biology. However, the targeted delivery of these systems remains a major challenge due to the large and chemically distinct nature of their components, including Cas protein or its base/prime editor fusions, guide RNA, and in some cases, DNA repair templates—which complicate packaging, stability, and cellular uptake. Additional hurdles arise from tissue and cell-type specificity, differential intracellular environments, variable editing efficiencies, and the persistent risk of off-target genome modifications. This review outlines the key challenges in the delivery of CRISPR technologies as well provides a comprehensive overview of both current and emerging delivery strategies, including viral vectors (adenovirus, adeno-associated virus, and lentivirus), non-viral physical approaches (microinjection, electroporation, ultrasound, and hydrodynamic tail-vein injection), and nanoparticle-based modalities (lipid and polymeric nanoparticles, gold nanoparticles, DNA nanostructures, and extracellular vesicles). We also discussed the diverse applications of CRISPR-Cas9 in gene therapy, immune cell engineering for cancer therapies, and agricultural innovation.

## Abstract 2

This figure illustrates the different delivery modalities used for CRISPR delivery and their applications in gene therapy, agriculture, and other emerging fields.

## Graphical abstract

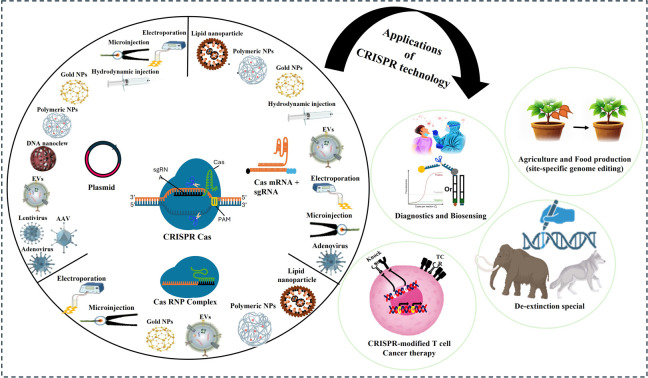

## 1 Introduction

Clustered regularly interspaced short palindromic repeats (CRISPR) and CRISPR-associated (Cas) systems were first identified as adaptive immune mechanisms in bacteria and archaea, providing sequence-specific defense against genetic elements such as bacteriophages, plasmids, and transposons.^1–3^ Early sequencing studies revealed that these systems capture fragments of invading DNA (known as spacers) and integrate them into CRISPR arrays (a repetitive loci interspersed with unique spacer sequences) that function as “heritable molecular memories” of past infections.^4,5^ Upon subsequent infections, the CRISPR array is transcribed to generate the CRISPR RNA (crRNA), which dictates target specificity, and the *trans*-activating CRISPR RNA (tracrRNA), which acts as a scaffold to recruit Cas9 nuclease. Together, the crRNA-tracrRNA duplex forms a ribonucleoprotein complex with Cas9 that scans for complementary foreign DNA, forms an RNA–DNA hybrid, and directs Cas9 to cleave the foreign DNA to eliminate the threat. This discovery established CRISPR-Cas as a programmable, nucleic-acid-guided defense system, in which DNA targeting can be reprogrammed simply by altering the guide RNA sequence.^6,7^ Because of its inherent modularity and the simplicity of its RNA-protein architecture, CRISPR-Cas9 rapidly emerged as a broadly applicable and powerful platform for precise genome manipulation.^8,9^

**Fig. 1:**
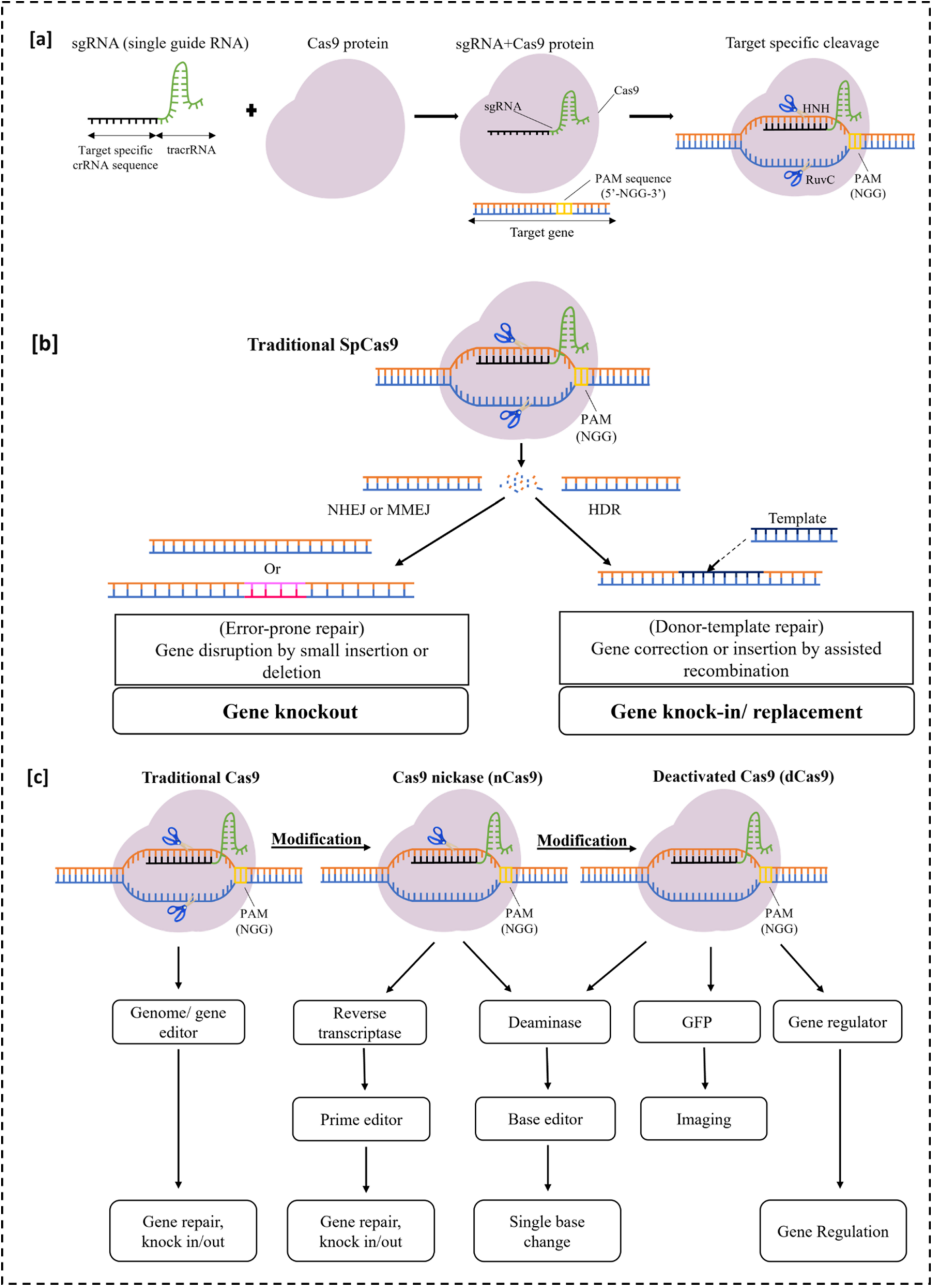
Mechanism of CRISPR-Cas9 genome editing. (a) The sgRNA forms a complex with Cas9 and guides it to the target DNA adjacent to a PAM sequence, enabling Cas9 to cleave both DNA strands. (b) The resulting double-strand break is repaired by either NHEJ/MMEJ, leading to indels and gene knockout, or by HDR using a donor template for precise gene knock-in or replacement. (c) Engineered Cas9 variants expand genome-editing functions: nCas9 (single-nickase) supports prime editing, while dCas9 (nuclease-deficient) enables base editing, imaging, and gene regulation through recruitment of effector proteins.

Building on this natural defense mechanism, researchers further simplified the CRISPR-Cas9 system by engineering the crRNA and tracrRNA into a single guide RNA (sgRNA) that directs the *Streptococcus pyogenes* Cas9 nuclease to a chosen genomic locus. Target recognition requires both Watson–Crick complementarity between the sgRNA spacer, the target DNA strand (TS) and the presence of a protospacer adjacent motif (PAM) on the opposite non-target strand (NTS) adjacent to the target site. PAM binding initiates local DNA unwinding and R-loop formation, allowing the sgRNA to pair with the target strand and properly orient Cas9's catalytic domains for DNA cleavage (Fig. 1). The HNH domain cuts the target strand, and the RuvC domain cleaves the non-target strand, generating a site-specific double-strand break (DSB).^6,7,10^ Cellular repair pathways, primarily non-homologous end joining (NHEJ) or homology-directed repair (HDR), then process the DSBs to produce targeted gene disruption, correction, or insertion.^11–15^ Together with these mechanistic insights, the demonstration by Emmanuelle Charpentier and Jennifer Doudna of programmable genome editing, and its broad applications, ultimately led to them being awarded the 2020 Nobel Prize in Chemistry.^16^

Advances in protein and RNA engineering have dramatically expanded the CRISPR toolkit beyond canonical DSB-mediated genome editing. Structure-guided mutagenesis of Cas9's catalytic domains such as mutation of the RuvC nuclease domain (D10A) or the HNH domain (H840A) generates nickase Cas9 (nCas9), which introduces single-strand breaks, while the combination of both mutations (D10A/H840A) yields catalytically inactive Cas9 (dCas9).^6,10^ These nuclease domain engineered backbones have enabled a broad suite of precision CRISPR technologies developed by David Liu.^17^ Fusing nCas9 to cytosine or adenine deaminases generated base editors that install targeted C → T or A → G conversions without making double-strand breaks.^18,19^ Importantly, approximately 60 percent of known pathogenic human single-nucleotide variants fall within these transition classes, underscoring the therapeutic potential of base editing.^20^ Prime editing further extends this concept by coupling nCas9 to an engineered reverse transcriptase and by redesigning the guide RNA into a prime-editing guide RNA (pegRNA), which both directs Cas9 to the target site and provides the encoded repair template. Prime editing can install all 12 possible base substitutions, including transition, transversion mutations, small insertions, small deletions, and their combinations, making it a versatile genome editing suite.^21^ In parallel, dCas9 has been adapted for gene regulation by tethering transcriptional activators or repressors to create CRISPRa and CRISPRi systems that modulate endogenous gene expression.^22–28^ dCas9 can also be fused to fluorescent proteins or paired with fluorescently labeled guide RNAs to allow real-time imaging of chromatin organization, genome movement, and transcriptional activity in living cells.^29–31^ Collectively, these engineered derivatives position CRISPR-Cas technology as a modular and versatile platform for targeted sequence modification, epigenomic control, and high-resolution visualization of genome function.^32,33^

The development of CRISPR-Cas9 genome editing has revolutionized both basic and applied scientific research by enabling rapid analysis of previously inaccessible questions. Of all scientific disciplines, medical science has witnessed the swiftest translation of CRISPR technologies.^34–37^ For example, CASGEVY (exagamglogene autotemcel) is the first FDA-approved CRISPR/Cas9 therapy for severe sickle cell disease and transfusion-dependent β-thalassemia (Table 1). This treatment involves editing the erythroid enhancer region of the *BCL11A* gene in a patient's own hematopoietic stem cells, thereby reactivating fetal hemoglobin expression.^38,39^ Numerous other CRISPR-based therapies are currently in clinical development, including NTLA-2001 for transthyretin amyloidosis, EDIT-101 for Leber congenital amaurosis, CTX110 and CTX130 as allogeneic CAR-T cell therapies, VERVE-101 and VERVE-102 for familial hypercholesterolemia, BEAM-101 for sickle cell disease using base editing, and PM359 for inherited chronic granulomatous disease. These ongoing trials underscore the rapid progression of CRISPR-based technologies in a decade from bench to bedside (Table 1).^40,41^ CRISPR-based diagnostics such as SHERLOCK and DETECTR further extend the translational impact of CRISPR-technologies by enabling rapid, point-of-care detection of pathogens and genetic variants.^42–45^ These diagnostic technologies used the collateral cleavage activity of Cas enzymes (Cas12 or Cas13) to enable highly sensitive SARS-CoV-2 detection during the COVID-19 pandemic.^46,47^

**Table 1:** Selected CRISPR-/base-editing/prime-editing clinical trials

Clinical trial/product (modality)	Target gene(s)/locus	Therapeutic indication	Clinical trial phase	Clinical trial identifier
Exagamglogene autotemcel (exa-cel/CTX001/Casgevy) CRISPR-Cas9 (*ex vivo*)	*BCL11A* erythroid enhancer	Sickle cell disease β-thalassemia	Phase 2/3 completed; FDA/EMA approved	NCT03745287, NCT03655678, plus long-term follow-ups
NTLA-2001 CRISPR-Cas9 (*in vivo*)	*TTR*	Hereditary transthyretin amyloidosis	Phase 1; phase 3 ongoing	NCT04601051, NCT06128629
EDIT-101 (AGN-151587) CRISPR-Cas9 (*in vivo*, subretinal)	*CEP290* IVS26 mutation	Leber congenital amaurosis type 10 (LCA10)	Phase 1/2	NCT03872479
CTX110 CRISPR-Cas9 allogeneic CAR-T	*TRAC*, *B2M* knock-out; anti-CD19 CAR insertion	B-cell malignancies (*e.g.*, LBCL)	Phase 1/2	NCT04035434
CTX130 CRISPR-Cas9 allogeneic CAR-T	CD70 CAR insertion (with edits in donor T cells)	T-cell lymphoma; clear-cell renal carcinoma	Phase 1/2	NCT04438083, NCT04502446
VERVE-101 *in vivo* adenine base editor (LNP)	*PCSK9*	Familial hypercholesterolemia/ASCVD	Phase 1b	NCT05398029
VERVE-102 *in vivo* base editor (GalNAc-LNP)	*PCSK9*	Familial hypercholesterolemia; premature CAD	Phase 1b	NCT06164730
BEAM-101 *ex vivo* adenine base-edited autologous HSPCs	*HBG1*/*HBG2* promoter (to derepress HbF)	Sickle cell disease	Phase 1/2	NCT05456880
PM359 (Prime-0101) *ex vivo* prime editing	*NCF1* (p47phox)	Chronic granulomatous disease (p47phox CGD)	Phase 1/2	NCT06559176

Beyond therapeutics, CRISPR technologies help accelerating the development of disease-resistant, climate-resilient, and high-yield crops.^48^ For example, editing the wheat susceptibility gene in Mildew resistance locus O (*MLO-B1*) with a 304 kb deletion produced powdery mildew-resistant wheat plants with the same yield as wild type.^49^ In livestock science, genome editing is enabling precise enhancement of commercially and biologically important traits including the animals with disease resistant traits.^50^ For example, editing the CD163 receptor in pigs or CD46 receptors in cows produced animals resistant to porcine reproductive and respiratory syndrome (PRRS) and bovine viral diarrhea virus (BVDV), respectively.^51,52^ Environmental biotechnology is also rapidly advancing, with CRISPR being applied to engineer microbial systems for improved bioremediation and sustainable bio-manufacturing.^53,54^ CRISPR-based gene driven strategies are under evaluation for population control and disease-vector suppression in regions burdened by insect-transmitted pathogens such as malaria and Lyme disease.^55–58^

**Fig. 2:**
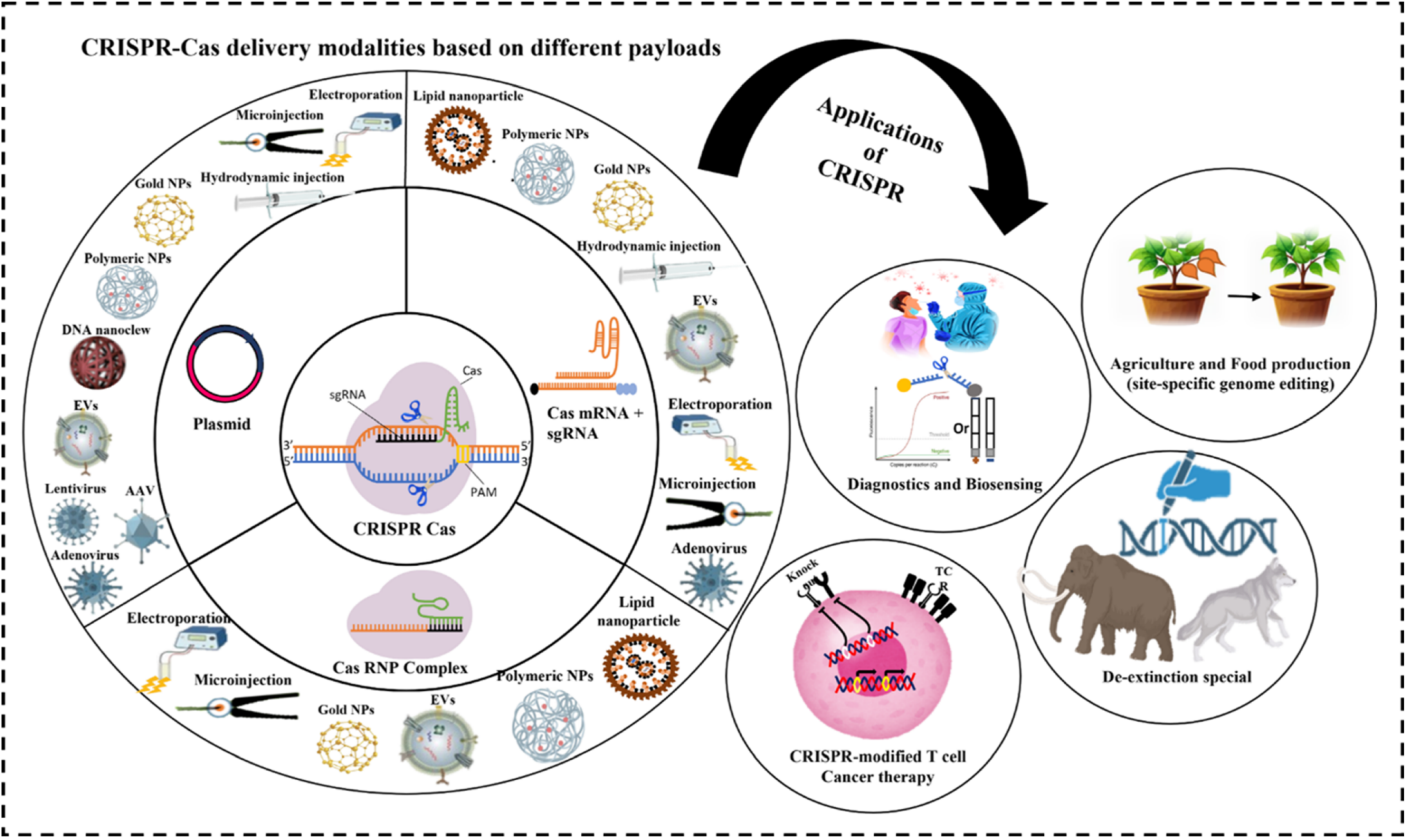
Schematic illustration of delivery platforms for CRISPR-Cas and their applications in healthcare and agriculture.

In this review, we discuss key challenges associated with the delivery of CRISPR technologies and illustrate how advances in viral, non-viral, and physical delivery strategies are addressing these limitations to improve the safety and efficiency of genome editing. Finally, we will highlight the use of delivery technologies in translational applications of CRISPR-based systems across gene therapy, agriculture, and other emerging areas (Fig. 2).

## 2 Different modalities for CRISPR-Cas9 delivery

Despite its transformative impact, conventional CRISPR-Cas systems face several fundamental limitations that restrict their broad translational applicability. A major constraint lies in the intrinsic size and structural complexity of CRISPR components. The widely used SpCas9 is a ∼160 kDa nuclease (∼10 nm), and its associated gRNAs add an additional ∼3–5 nm; together these components far exceed the permeability limits of cellular membranes, which typically permit diffusion of small molecules such as glucose (∼1 nm).^59^ This size mismatch underscores the difficulty of achieving efficient tissue and cell-specific delivery, particularly *in vivo*, where physiological barriers, immune clearance and extracellular matrix density further impede transport. Moreover, CRISPR knockout (CRISPRko) strategies rely on endogenous DNA repair pathways to resolve Cas9 induced DSBs, often suffer from variable cutting efficiencies and produce heterogeneous populations of cells.^60^ DSBs can also trigger genotoxic stress, cytotoxicity or apoptosis.^61,62^ These issues can amplify in cancer cells where excessive DSB formation may generate false-positive phenotypes or selective cell loss. In parallel, off target editing, unintended chromosomal rearrangements and immunogenicity of bacterial Cas proteins remain major safety concerns for clinical translation.^63,64^

Conventional methods to regulate Cas9 activity, including systems that modulate nuclease half-life, are often slow, inefficient and reliant on large (>230 amino acids) regulatory modules or expensive chemical inducers with poor pharmacological profiles, limiting their suitability for *in vivo* use.^65^ These biological constraints are compounded by persistent delivery challenges: the field still lacks universal, safe, and efficient vectors capable of achieving precise spatiotemporal control, minimizing immune responses and providing mechanisms such as kill switches for reversible or self-limiting genome editing.

**Fig. 3:**
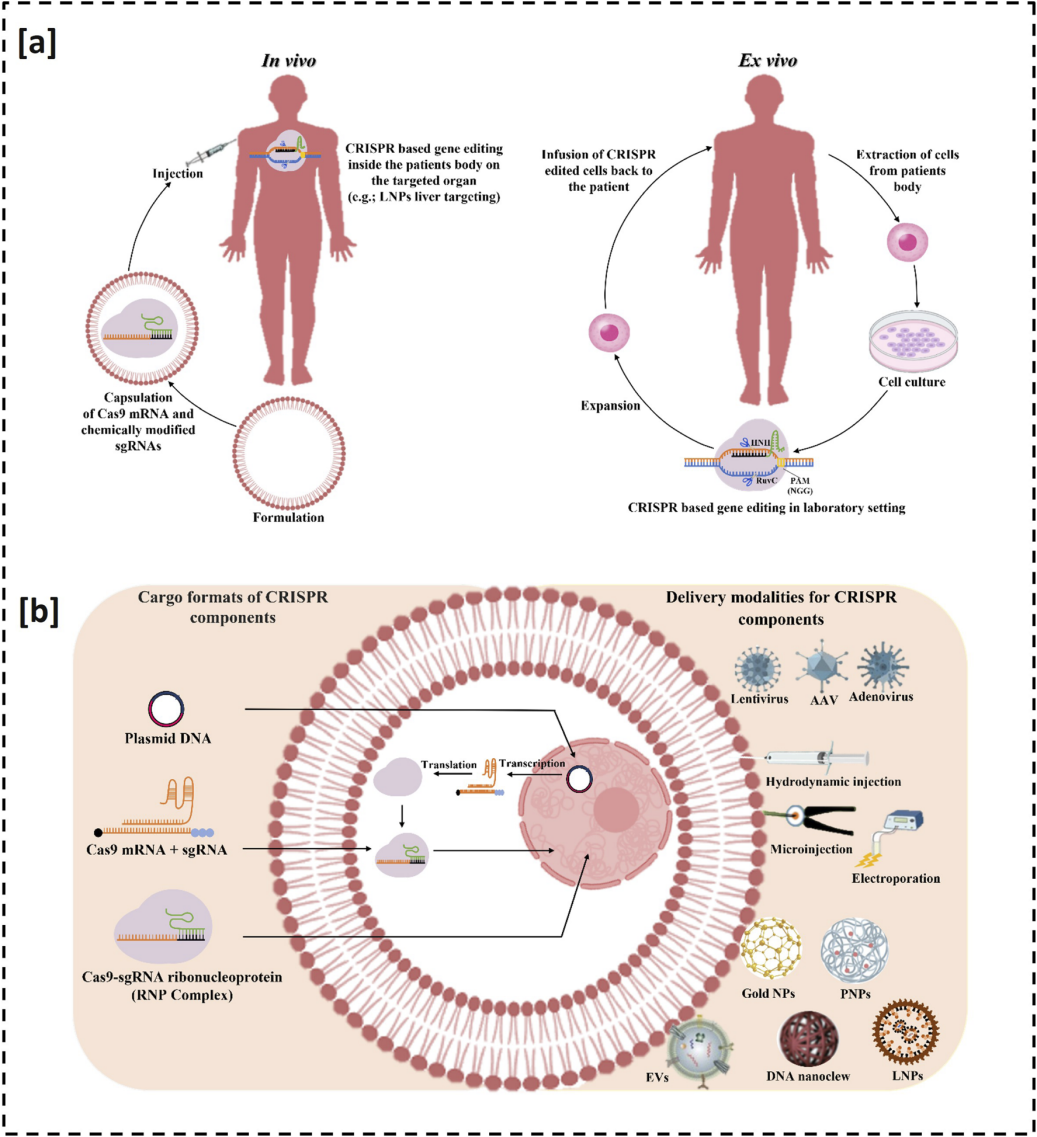
Genome editing *via* the CRISPR-Cas9 system: (a) two primary modes of CRISPR-Cas9 delivery—*in vivo* and *ex vivo*. (b) Delivery of three main cargo formats of CRISPR components through different delivery systems such as viral delivery systems, non-viral and physical delivery systems.

Although viral vectors, non-viral nanocarriers and physical delivery strategies each offer partial solutions (Fig. 3), they also introduce trade-offs in cargo capacity, tissue tropism, toxicity and manufacturability.^66–68^ For example, adeno-associated virus (AAV) vectors remain among the most reliable systems for *in vivo* delivery of base editors; however, their prolonged transgene expression increases the risks of off-target editing and vector integration, raising concerns about genotoxicity and carcinogenesis. These limitations highlight the need for next-generation CRISPR platforms that emphasize precision, safety and controllability to fully realize the therapeutic potential of genome editing. For example, the challenges associated with AAV have driven the development of alternative strategies such as direct delivery of base editor ribonucleoproteins (RNPs), which exhibit shorter intracellular halftimes and reduced off-target activity and emerging virus-like particle platforms that can deliver RNPs without introducing a viral genetic material.^69^ In the following sections, we discuss different delivery modalities explored for CRISPR-based genome engineering with their advantages, disadvantages and case studies.

### 2.1 Viral delivery systems

Recombinant viral vectors engineered to deliver therapeutic genes to diseased tissues by harnessing the natural ability of many viruses to introduce foreign genetic material into host cells.^70^ Viral vectors have been in development for over 30 years to deliver genetic treatments like various nucleic acid-based therapeutics; more than 1000 viral gene therapy clinical trials have been conducted and some therapies have even been approved for clinical use. While there are concerns about their safety and the risk of causing unwanted mutations, viral vectors remain one of the most effective methods for getting plasmid-based genetic material into mammalian cells, both in the lab and in living organisms. Because of this, they have become a popular tool for delivering CRISPR-Cas9 gene editing technology into mammalian cells.^68^ The most used viral vectors are adeno-associated virus (AAV), lentivirus, and adenovirus^71^ (Table 2).

**Table 2:** Different viral delivery systems

Vector type	Package limitation	Superiority	Advantages	Disadvantages	Application	References
Adeno-associated virus (AAV)	4.5 kb	Low immunogenicity, stable transgene expression	High infection efficiency, broad cell tropism	Low packaging capacity, difficulty in production	Treatment of lipoprotein lipase deficiency patients, edit the *VEGFR2* gene in retinal endothelial cells within a mouse model, single-cell CRISPR screening in mouse brain	72–74
Lentivirus (LV)	8 kb	Large packaging capacity, low cell cycle tendency	High infection efficiency, large packing size, long-term gene expression, persistent gene transfer	Long lasting expression of Cas9, potential for insertional mutagenesis	Epigenome editing targets cytokines (TNF-α, IL-1β) in the intervertebral disc to treat degenerative disc disease and chronic low-back pain, gene editing tool delivery with a gag-only strategy, vaccine development	75,76
Adenovirus (AV)	>8 kb	Large packaging capacity, no integration to the host genome	Risk of off-target effects, strong immunogenicity, limited cargo capacity for gene constructs, limited reusability, high delivery efficiency	High immunogenicity, difficult scale production	Induces targeted mutations in a wide range of human cells, selective targeting of vascular endothelial cells *in vivo*, cancer treatment, gene therapy	77,78

#### 2.1.1 Adeno-associated virus (AAV) delivery system

Adeno-associated virus (AAV) is among the most used viral vectors for gene delivery due to its wide range of serotype specificity, capacity to infect both dividing and non-dividing cells, and advantageous safety profile.^79^ AAV is non-pathogenic, replication-defective, and does not integrate into the host genome, which contributes to its low immunogenicity in humans.^80^ These characteristics have made AAV an interesting platform for *in vivo* gene delivery applications and its ability to deliver transgenes episomally also provides a safer alternative for transient gene expression without the risk of genomic disruption.^71,81^ AAV can be used to deliver CRISPR/Cas9 components such as Cas9, sgRNAs, and/or donor templates into cells by transduction. AAV is a small (∼20 nm), non-enveloped, icosahedral parvovirus, which carries a single-stranded DNA genome of approximately 4.7 kb and has over 200 different molecularly engineered or naturally occurring serotypes with diverse tissue tropisms. However, its limited packaging capacity presents a challenge for delivering larger constructs like full CRISPR-Cas9 systems.

To overcome this, AAV have evolved strategies like overlapping open reading frames (ORFs) to increase coding capacity without expanding genome length.^82^ The European Medicines Agency (EMA) approved Glybera (alipogene tiparvovec) as a first AAV-based gene therapy drug in the European Union in 2012. Glybera is an adeno-associated viral vector engineered to express lipoprotein lipase gene into the muscle for the treatment of lipoprotein lipase deficiency patients.^83^ Despite its advantages, *in vivo* genome editing still faces challenges related to efficiency and safety. A targeted approach involves using the endothelial-specific intercellular adhesion molecule 2 (pICAM2) promoter, which drives expression of SpCas9, ensuring targeted expression of the Cas9 enzyme within vascular endothelial cells that are efficiently transduced by AAV1; this system is effective effectively used to edit the VEGFR2 gene in retinal endothelial cells in a mouse model of oxygen-induced retinopathy, showcasing its potential for targeted vascular genome editing.^84^

#### 2.1.2 Lentivirus (LV) delivery system

Lentiviral vectors are commonly used in gene therapy due to their ability to provide long-term expression of the inserted genes with low immune response (mild immunogenicity). They are highly efficient at infecting cells, even those that are not actively dividing.^68^ They don't require the target cells to divide for integration into the host genome, which broadens the range of cells that can be effectively targeted by retroviral gene transfer. To create a lentivirus, two types of plasmids are needed: (1) packaging plasmids, which carry the instructions for making viral particles, including structural proteins and enzymes. (2) Plasmids that carry the foreign genetic material, such as the Cas9 gene or sgRNA cassettes, which are essential for genome editing.^85^ Lentivirus-based delivery of CRISPR-Cas9 is a powerful method for conducting function-based screening in mammalian cells and creating knockout animal models. The delivery of a genome-scale CRISPR-Cas9 knockout (GeCKO) library, which targets 18 080 genes and includes over 64 000 unique guide sequences, allows for both positive and negative selection screening in human cells and improving whole-genome screens.^86^ The feasibility of lentiviral CRISPR epigenome editing as a targeted gene therapy approach to reduce harmful inflammation, particularly by cytokines such as TNF-α and IL-1β within the intervertebral disc (IVD), which drive the Degenerative disc disease (DDD), the leading cause of chronic low-back pain and disability, has been demonstrated. While TNFR1 suppression proved effective (reducing NF-κB activity, cell death, and pro-inflammatory gene expression following cytokine challenge), IL1R1 targeting presents challenges due to feedback mechanisms, as some patient samples showed rebound expression driven by IL-1β. Additionally, RNA sequencing identified two key transcription factors, IRF1 and TFAP2C, as central regulators of inflammatory signaling in IVD cells. Still, this method opens the door for novel therapies that address the root inflammatory drivers of DDD.^87^

#### 2.1.3 Adenovirus (AV) delivery system

Adenoviruses are non-enveloped and non-integrating viruses with a double stranded genome of approximately 36 kb.^88^ Compared to retroviral and lentiviral vectors, AV can infect both dividing and non-dividing cells and offer greater flexibility in accommodating larger exogenous DNA sequences due to their large genome size. Using adenoviral vectors to deliver RNA-guided CRISPR/Cas9 nuclease (RGN) complexes successfully induces targeted mutations in a wide range of human cells.^89^ Researchers used a novel approach by engineering the capsid of a chimpanzee-derived adenovirus isolate (SAd36) to modify its cellular tropism. Specifically, a myeloid cell-binding peptide (MBP) was incorporated into the capsid, enabling selective targeting of vascular endothelial cells *in vivo*. This refers to an engineered SAd36. MBP vector, which has shown promise as a single-dose intravenous delivery system capable of achieving precise genome editing in pan-endothelial cells across multiple organs, including the spleen, brain, and kidneys in mice, offering an attractive non-hepatic route for expressing serum proteins, which could be transformative for treating various inherited genetic disorders.^90^ Beyond targeted delivery, using a nonhuman primate adenovirus also provides a significant advantage by helping to evade pre-existing immunity commonly seen with human adenovirus-based therapies.

### 2.2 Non-viral and physical delivery systems

Viral delivery systems with high issues related to safety and the risk of causing unwanted mutation there is need to use physical delivery system^91,92^ (*e.g.* microinjection, ultrasound, hydrodynamic tail-vein injection and electroporation) and non-viral delivery system^93–95^ (*e.g.* nanoparticles) are relatively simple to carry out but are primarily suited for *in vitro* applications (Table 3). Nanotechnology refers to the engineer nanomaterials at the nanoscale, generally within the range of 1–100 nm such as carbon nanotubes, gold nanoparticles and quantum dots.^96^ In biology and medicine, nanoparticles can be used for drug delivery, biosensing, diagnostic imaging and even gene editing. Their unique properties of nanoparticles, such as their size and reactivity, make them ideal candidates to interact with biological systems of living cells in ways that allow for visualizing, high-resolution imaging and monitoring of cellular processes at the molecular level, and their surface properties can be engineered to target specific cells or tissues. It provides a promising solution to challenges in sensitivity, resolution, real-time tracking and efficiency of delivery. The intersection of CRISPR-Cas9 technology and nanotechnology particularly advanced nanomaterials allows for the engineering of nanoscale materials that enable real-time tracking and high-resolution imaging of the CRISPR-Cas9 gene-editing process. This interdisciplinary approach promises breakthroughs in molecular biology, agriculture, precision medicine, and therapeutic development.^97,98^

**Table 3:** List of physical delivery systems

Delivery system	Advantages	Disadvantages	Application	References
Microinjection	Dosage controllable, direct delivery, highly specific, suitable for all strategies of CRISPR-Cas9	Labor-intensive, low throughput, potential for cell damage, time consuming, *in vitro* only	Animal model generation, plant genome editing, precision editing in oocytes and zygotes	99
Ultrasound	Non-invasive, potential for targeted and controlled delivery	Limited tissue penetration, potential for tissue damage, requires specialized equipment	Potential for non-invasive gene therapy, cancer treatment, stem cell engineering with minimal damage	100 and 101
Hydrodynamic tail-vein injection (HTVI)	Easy to operate, low price	Traumatic to tissue, low specificity, can cause hepatocyte membrane disruption and cellular stress, reducing gene delivery efficiency	*In vitro* and *in vivo* gene editing efficiency, efficient liver-targeted gene delivery using lentiviral vectors through HTVI with reduced off-target distribution to other organs	102 and 103
Induced transduction by osmocytosis and propanebetaine (iTOP)	Reliable method for delivery of cas9 protein and sgRNA, virus-free and non-integrative	Unsuitable for *in vivo* applications, high salt concentrations required	Primarily utilized in *ex vivo* gene editing applications, highly efficient transduction of native proteins, efficient manipulation of a wide variety of primary cell types	104
Electroporation	Easy to operate, suitable for any cell type, high transfection efficiency, suitable for all strategies of CRISPR-Cas9 and can be used *in vitro* and *in vivo*	Potential for cell damage, affects cell viability, requires specialized equipment, *in vitro* only and nonspecific transfection	*Ex vivo* and *in vivo* delivery (*e.g.*, cancer immunotherapy)	105 and 106

#### 2.2.1 Microinjection

Microinjection is a technique which involves the precise delivery of foreign molecules, such as the CRISPR-Cas9 system, directly into embryonic or other living cells using a fine glass micropipette under a microscope with high reproducibility. Which mainly used it laboratory to deliver exogenous protein or nucleic acids into single cells and to modify genomic content in the cells of rabbits,^106^ zebrafish,^107^*Ciona intestinalis*,^108^*C. elegans* (*via* CRISPR/Ca9 system)^109^ and *Aedes aegypti*.^110^ It can create knockout mice using several CRISPR/Cas9 systems into freeze-thawed fertilized oocytes.^111^ It also serves as an essential technique for conducting embryological and genetic analysis, particularly using medaka and zebrafish as a model genetic organism in a laboratory for example Medaka a small freshwater egg-laying fish with transparent embryos that develop externally serves as an ideal model organism, one-cell embryos can be microinjected with 50–150 pg plasmids^112^ where microinjection technique plays a vital role in the delivery of cell-tracers for labelling cells, mRNAs or antisense oligonucleotides for overexpressing and knocking-down genes of interest, and DNAs for creating transgenic lines.^113^

#### 2.2.2 Ultrasound

Ultrasound is a promising physical delivery system for targeted, controlled and non-invasive delivery of foreign molecules. For example, an ultrasound-controlled CRISPR/Cas9 delivery system, known as HMME@Lip-Cas9, was carefully engineered to precisely knock down NFE2L2 (a gene that is immediately activated during SDT and can limit its effectiveness).^114^ While sonodynamic therapy (SDT) is a promising non-invasive clinical treatment for hepatocellular carcinoma (HCC), its efficacy is often hindered by the cancer cells' antioxidant defense mechanisms and the high tissue-penetration depth of ultrasound. By targeting and suppressing NFE2L2, which reduces oxidative stress, the HMME@Lip-Cas9 system helps overcome these limitations enhancing the production of reactive oxygen species (ROS) which is yielded in abundant amount by hematoporphyrin monomethyl ether (HMME) system to damage cancer cells under ultrasound irradiation and thereby improving the therapeutic outcomes of SDT79. Another team has demonstrated the potential of focused ultrasound (FUS) combined with microbubble-mediated delivery to introduce CRISPR-Cas9 single-guide RNA (sgRNA) ribonucleoproteins (RNPs) into human induced pluripotent stem cells (hiPSCs) *in vitro*.^115^ This approach offers a promising gene-editing platform due to its high on-target efficiency, rapid action, and reduced risk of insertional mutagenesis compared to other CRISPR-Cas9 delivery formats. In conclusion, hiPSCs could serve as a valuable model system in gene therapy research, particularly for cardiovascular applications. For instance, hiPSC-derived cardiomyocytes can be used to study and potentially treat inherited disorders such as hypertrophic cardiomyopathy, a condition that currently lacks effective therapeutic strategies and is an ideal candidate for CRISPR-Cas9 based interventions. However, despite these advances, existing *in vivo* delivery approaches for Cas9 still carry the risk of triggering unwanted immune responses, underscoring the need for safer, non-invasive delivery systems like FUS42. A combination of pH-responsive lipid-polymer hybrid nanoparticles (PLPNs) and ultrasound-mediated microbubble destruction (UMMD) has been developed for the high-performance delivery of a dCas9-based CRISPR Interference (CRISPRi) system.^116^ This approach enables efficient, tumor-specific gene repression by silencing target genes without inducing detectable off-target effects.

#### 2.2.3 Hydrodynamic tail-vein injection (HTVI)

HTVI method has emerged as one of the simplest and most effective techniques for delivering nucleic acids in animal models.^117^ Gene delivery *via* this method requires only an expression vector dissolved in physiological saline, although it lacks protective components in its formulation.^118^ For example, a CRISPR/Cas9-containing vector can be dissolved in physiological saline and rapidly injected into rodents *via* the tail vein at a volume equal to 8–10% of the animal's body weight applying high pressure during administration.^119^ Due to the rapid injection of a large volume of solution, sudden influx creates hydrodynamic pressure, which temporarily opens pores in the membranes of endothelial cells and physically displaces existing blood components including nucleases, allowing the nucleic acids or proteins to enter cells quickly and efficiently, minimizing degradation and maximizing cellular uptake. The rapid flow of saline causes liver expansion, facilitating fluidic transduction of hepatocytes and enhancing gene delivery to the liver. Due to its efficiency and simplicity, HTVI is widely used for *in vivo* delivery of various biomolecules, including plasmid DNA, proteins, small interfering RNA (siRNA), and even cancer cells.^120^ The ease of this method has positioned it as a leading technique for rapidly establishing liver cancer and other disease models using plasmid-based CRISPR-Cas9 systems.

#### 2.2.4 Induced transduction by osmocytosis and propanebetaine (iTOP)

The iTOP is considered a reliable and efficient method for delivering CRISPR components either as separate Cas9 protein and sgRNA (intracellular delivery) or as ribonucleoprotein (direct delivery) complexes into various human cells, including primary T cells, induced pluripotent stem cells (hiPSCs), Jurkat, ARPE-19, HEK293 cells.^121^ This method has demonstrated CRISPR/Cas9 gene-editing efficiencies from 40% to 95% depending on the cell type, highlights the potential of iTOP-mediated delivery for effective genome engineering.^121^ Importantly, cell viability remains high after treatment, typically between 70% and 95%. CO-delivering Cas9 protein and sgRNA together into human embryonic stem cells has achieved gene-editing efficiencies of up to 26% following two rounds of administration.^104^ This technique has been successfully used to introduce RNPs into various types of primary cells. The process involves using a hyperosmotic buffer containing sodium chloride and propanebetaine, a specialized transduction agent, which triggers macro-pinocytosis and allows the cell to absorb the genetic material effectively (cargos).^68^ The iTOP technique permits efficient intracellular delivery of macromolecules and facilitates the high-efficiency transduction of native proteins without the need for a transduction peptide sequence. It also supports effective manipulation of a wide range of primary cell types. However, despite its advantages *in vitro*, iTOP is not suitable for *in vivo* applications because the Cas9 protein is only soluble in the high salt concentrations required by iTOP. In contrast, other methods such as electroporation, cell-penetrating peptides (CPPs), and cationic lipid-mediated delivery have demonstrated effective protein-based genome editing both *in vitro* and *in vivo*.^122^

#### 2.2.5 Electroporation

For the past 30 years, electroporation has been widely used to introduce DNA into cultured mammalian cells, yeast and bacteria. Significant advancements have been made during this period including its application in cell fusion, delivery of chemotherapeutic agents to cells and tissues and *in vivo* models like rodents. In this method, electrical fields use to destabilize the cell membrane so that impermeable macromolecules such as proteins or nucleic acids can enter the cytoplasm.^123^ Electroporation is utilized to transport proteins, mRNA and nucleic acids into mammalian cells with the help of different delivery payloads. Electroporation could rapidly and effectively disrupt the lipid bilayer, leading to an immediate increase in membrane permeability.^124^ This enhanced membrane damage facilitates transmembrane drug transport, creating a favorable environment for efficient delivery; however, limitations include low plasmid DNA integration efficiency (approximately 0.01% of target cells), significant cell death, the need for specialized equipment, nonspecific transfection, and its primary applicability to *in vitro* systems.^125,126^

A novel method called Easy Electroporation of Zygotes (EEZ) offers a highly efficient way to generate CRISPR/Cas9 transgenic mice while also reducing the number of animals needed.^127^ This approach provides a faster and less invasive alternative to traditional pronuclear injections for genome editing in mice. EEZ is particularly well-suited for introducing targeted mutations in C57BL/6 mice by electroporating intact zygotes using a standard electroporator, synthetic CRISPR/Cas9 components, and minimal technical complexity. Unlike earlier protocols that require specialized electroporation equipment or harsh pre-treatment of zygotes often at the expense of embryo viability EEZ minimizes physical damage, resulting in improved embryo development. However, genome editing success has been reported in up to 100% of live-born offspring using this method, making it a practical and accessible tool for researchers.^128^ Mutant mice can be generated in a single step by directly delivering CRISPR/Cas9 genome editing components into mouse zygotes *via* electroporation. This approach helps to overcome the limitations of traditional low-throughput methods and enables the efficient recovery of live mice carrying targeted mutations through both nonhomologous end joining (NHEJ) and homology-directed repair (HDR).^129^

Nucleofection-based CRISPR/Cas9 genetic engineering has been proven as an effective and rapid method for creating mutant CD8^+^ T cells from both naïve and *in vitro*-activated primary mouse cells. This technique allows for efficient genetic modification without significantly compromising the cells' *in vivo* functionality. Experimental testing of their immune responses revealed that nucleofected naïve CD8^+^ T cells retained antiviral immune activity comparable to that of non-nucleofected cells. While nucleofection of *in vitro*-activated CD8^+^ T cells resulted in slightly reduced expansion and survival shortly after adoptive transfer, it also led to a more noticeable contraction phase over time.^130^ Researchers have showed that *in vivo* electroporation of morpholino oligonucleotides targeting fgfr1 and msxb proteins offers a practical and effective method for protein knockdown during zebrafish fin regeneration (a widely used model organism for studying tissue regrowth). This technique led to reduced fin outgrowth, with Fgfr1 knockdown mimicking the effects of a known Fgfr1 inhibitor. Additionally, the approach provided direct evidence of msxb's functional role in caudal fin regeneration. Interestingly, while knocking down Fgfr1 disrupted the expression of msxc in the regenerative blastema, msxb knockdown did not indicate that this method can also be used to investigate genetic relationships and epistasis within regeneration pathways. Thus, this convenient reverse genetic tool enables researchers to rapidly assess the role of genes expressed during fin regeneration, screen candidate genes for functional importance and map genes to the molecular pathways that drive regeneration.^131^ (Table 4). represents the non-viral delivery systems.

**Table 4:** List of non-viral delivery systems

Delivery system	Advantages	Disadvantages	Application	References
Lipid nanoparticles (LNPs)	Easy to prepare, low cost, high transfection efficiency, biocompatible, biodegradable, safe and suitable for all strategies of CRISPR-Cas9	Potential immunogenicity, off-target effects, possible degradation in biological fluid before reaching target, low delivery efficiency, specific cell tropism	*In vitro* and *in vivo* gene therapy, (*e.g.*, lung and liver editing, epigenetic editing into solid tumors, cancer therapy, potential for neurodegenerative disease treatment)	132 and 133
Polymer nanoparticles (PNPs)	Easy to prepare, high stability, controlled release, biocompatible, safe and suitable for all strategies of CRISPR-Cas9	Lower transfection efficiency, some PNPs may induce cytotoxicity, low delivery efficiency	*In vitro* and *ex vivo* stem cell engineering, controlled release editing for prolong gene expression, chronic disease treatment, (*e.g.*, genome-editing for CNS disease, *etc*)	134 and 135
Gold nanoparticles (AuNPs)	High delivery efficiency, easy surface functionalization, efficient delivery of CRISPR RNPs, mainly lower cytotoxicity	Limited capacity for delivery large gene-editing payloads, expensive production, potential long-term accumulation and toxicity concerns *in vivo* at high concentration	Potential for non-invasive CRISPR delivery to brain, cellular imaging and tracking and minimal off-target editing, precise gene editing in cancer therapy, (*e.g.*, breast cancer)	136
DNA nanostructure	Stable assembly, multiple editing, well histocompatibility, biocompatible, controllable size and architecture	Assembly is complicated, stimuli responsive release, requires modification of template DNA	*In vivo* as well as *in vitro* (*e.g.*, cancer treatment, gene therapy, *etc*)	137
Extracellular vesicle (EVs)	Biocompatible, low immunogenicity, efficient cellular uptake, target delivery, high delivery capacity, cross biological barriers	Limited loading capacity, potential off-target effects, unpredictable biodistribution as it may accumulate in different organs	Gene therapy, neurological disease treatment, cancer therapy, immune cell engineering, stem cell and regenerative medicine (*e.g.*, gene editing livestock gametes and embryos, cardiac repair)	138 and 139

#### 2.2.6 Lipid nanoparticles (LNPs)

Lipid nanoparticles (LNPs) are a popular choice for delivering nucleic acids, as they help protect them from breaking down by enzymes.^140^ They work by forming complexes with negatively charged nucleic acids and positively charged lipids through electrostatic interactions. These complexes are then taken up by cells through endocytosis. LNPs have been successfully used to deliver mRNA, plasmids, and small interfering RNA (siRNA), showing good results in both preclinical tests and clinical trials for RNA-based therapies. However, when it comes to delivering CRISPR/Cas9 plasmids, LNPs still more efficiency needed for clinical applications.^141^ Their delivery of primary cells and *in vivo* models remains a challenge. But by modifying the lipid nanoparticle system, there is a lot of potential to improve their efficiency. LNPs are also being explored to deliver CRISPR/Cas9 for therapeutic purposes or to create knockout animal models.

Researchers have combined two powerful tools: CRISPR/Cas9 gene editing and a targeted nanoparticle-based delivery system, supported by focused ultrasound (FUS) technology to construct lipid-polymer hybrid nanoparticles for delivery of the CRISPR/Cas9 system.^142^ In combination with focused ultrasound-microbubbles (FUS-MBs), this platform enables the opening of the blood–brain barrier (BBB) and knock down of MGMT (a gene known to drive resistance to temozolomide drug). The MBs-LPHNs-cRGD delivery system holds promise as an effective alternative method for targeted gene therapy in the treatment of glioblastoma (GBM) which is one of the toughest challenges in oncology. It has the potential to treat a wide range of genetic disorders, including cancers, but delivering it safely and effectively, especially within the central nervous system (CNS), remains a significant challenge due to the lack of reliability *in vivo* targeting systems.

#### 2.2.7 Polymer nanoparticles (PNPs)

Polymer nanoparticles are increasingly used to deliver various cargoes nucleic acids like plasmid DNA, mRNA and RNPs.^143,144^ In a recent study researchers revealed a new class of self-assembling carboxylated branched poly(β-amino ester) (PBAE) polymer-based nanocarriers has shown great promise as a flexible and efficient system for intracellular protein delivery directly into the cytosol of the cell even in the presence of 10% serum.^145^ Beyond protein delivery, these nanoparticles have demonstrated strong potential in CRISPR-based gene editing, achieving over 75% gene knockout and up to 4% gene knock-in across a variety of cell types.^145^ In preclinical studies, a single intracranial injection of just 3.5 pmol of CRISPR-Cas9 ribonucleoproteins (RNPs) encapsulated in these nanoparticles led to significant gene editing in mouse glioma brain tumors, highlighting their potential for *in vivo* therapeutic applications and future advancements in both research and clinical applications.

#### 2.2.8 Gold nanoparticles (AuNPs)

Gold nanoparticles (AuNPs) are gaining attention as a promising tool for delivering CRISPR/Cas9 ribonucleoprotein complexes (RNPs), due to their ability to easily bind with sulfhydryl (–SH) groups.^146^ This property enables precise control over their surface charge and hydrophilicity. To further enhance delivery, researchers have coated AuNPs with positively charged peptides, allowing them to electrostatic adsorption of Cas9 proteins.^147^ Research team have developed gold nanoparticles modified with cationic arginine to form nano-assemblies capable of simultaneously delivering sgRNA and Cas9 protein into cells.^148^ In another study, Cas9 proteins engineered with a glutamate peptide tag, along with sgRNA, are assembled with AuNPs to create highly efficient nanoparticles. This method has achieved over 90% delivery efficiency and around 30% gene-editing efficiency across multiple cell types.^148^ Its cholesterol-dependent membrane fusion entry sets this system apart from bypasses traditional cellular endocytosis pathways and potentially explaining its exceptional delivery performance. Researchers have developed a gold nanoparticle (AuNP)-based, lipid-encapsulated, laser-activated delivery system by attaching a TAT peptide with a C-terminal cysteine to the surface of the nanoparticles loaded with Cas9 protein. This design enabled the nanoparticles to be administered through intravenous injection, which presents a powerful and targeted approach for CRISPR/Cas9 gene editing, showing significant potential in cancer therapy and other genetic disease treatments.^149^ While this platform has shown strong promise for transient gene editing *in vitro*, its effectiveness in human primary cells, such as lymphoma cells, remains to be explored.

#### 2.2.9 DNA nanostructure

DNA has been engineered as programmable DNA nanostructures for various biomedical applications including bioimaging, sensing, stem cell therapy and targeted drug delivery.^150,151^ One of the key benefits of DNA is that its sequence can be precisely controlled, and its interactions are predictable, making it possible for DNA to naturally assemble into complex nanostructures. DNA is inherently biocompatible and water-soluble. Moreover, it can be easily synthesized using commercial synthesizers. Recently, DNA has emerged as a promising material for constructing nanostructures with potential applications in biotechnology and healthcare.^152^ For example, Qu *et al.* developed an innovative delivery and imaging system for near-infrared (NIR) light-controlled, non-invasive protein release using DNA as a template.^153^ Traditionally, creating DNA nanostructures has involved using Watson-Crick base pairing of small DNA fragments, which is a complicated process and requires a large amount of DNA. However, recent advancements like rolling circle amplification (RCA) have made this process much simpler.^154^ Sun *et al.* developed a new type of self-assembling, yarn-like DNA nanoclews through rolling circle amplification (RCA) for delivering CRISPR/Cas9 ribonucleoproteins (RNPs) in both *in vitro* and *in vivo* settings for genome editing.^154^ These biologically inspired nanocarriers are designed to transport both the Cas9 protein and its single guide RNA (sgRNA) directly into human cell nuclei, enabling precise gene editing while maintaining cell viability.

When the nanoclew DNA sequence shared partial complementarity with the sgRNA guide sequence it shows the highest gene editing efficiency, suggesting a useful design principle for optimizing delivery. This method offers a flexible and efficient platform not only for CRISPR-Cas9 but also for transporting other DNA-interacting proteins or functional nucleic acids in a similar fashion.^155^ For example, Francesca Miceli *et al.* developed MAIGRET (Molecular Assay based on antibody-Induced Guide-RNA Enzymatic Transcription), a two-step CRISPR-powered immunoassay to detect specific antibodies and antigens with high sensitivity using cell-free RNA transcription. In this process target antibody binds and triggers the transcription of a guide RNA, which activates Cas12a to cleave an optically labelled hairpin DNA reporter (fluorescent reporter), which leads to a measurable fluorescence signal. It can detect even complex samples like antibodies in the low picomolar range and can support antigen detection using a competitive format. Though slightly less sensitive than other methods (like-UCAD), it offers better signal control and broader adaptability with potential applications in diagnostics and could even be used in future gene-based therapies.^156^

#### 2.2.10 Extracellular vesicles (EVs)

Extracellular membrane-bound vesicles (EVs) possess a high delivery capacity, enabling the transport of various cargoes such as plasmid DNA, mRNA, and ribonucleoproteins (RNPs).^157^ They can cross biological barriers like the blood–brain barrier (BBB), exhibit high biocompatibility, low immunogenicity, and efficient cellular uptake, and offer targeted delivery for applications in gene therapy, neurological disorders, cancer treatment, immune cell engineering, and regenerative medicine.^158,159^ However, use of EVs is limited by potential off-target effects and unpredictable biodistribution, as they may accumulate in non-target organs.^160^ Exosomes naturally cross biological barriers and have minimal toxicity, making them an attractive option for CRISPR delivery.^161^ Surface modifications can enhance targeting abilities, but challenges remain in loading large nucleic acids and ensuring safe release. Further research is necessary to improve exosome-based CRISPR/Cas9 delivery for clinical applications.

## 3 Application of CRISPR-Cas9 technology across different areas

### 3.1 CRISPR-based diagnostics

In the process of CRISPR based diagnosis the guide RNA (gRNA or sgRNA) finds a complementary target sequence in DNA or RNA, then Cas protein cleaves the target recognized by guide RNA and ensures initial detection known as *cis*-cleavage. After binding the target in *cis*, many Cas enzymes become activated and begin cutting other nearby non-target nucleic acids indiscriminately, known as *trans*-cleavage, which is used as a signal amplification tool in which the *trans*-cleavage behavior can be directed toward specially designed reporter molecules—usually short single-stranded DNA or RNA tagged with a fluorophore and a quencher. When the *trans*-cleavage happens, the fluorophore is separated from the quencher, leading to a visible fluorescent signal. This engineered system allows researchers to toggle between target-specific (*cis*) cleavage and non-specific (*trans*) cleavage activity, by adjusting factors like guide RNA design, Cas protein variants, and reaction conditions which helps scientist to optimize the sensitivity, specificity, and timing of the signal generation. This activity is very important to avoid false positives, enhance signal clarity, and ensure accurate detection even at very low concentrations of the target DNA or RNA.

The CRISPR-Cas system has recently gained popularity as a diagnostic tool in various clinical and point-of-care applications due to its ability to specifically target genes and cleave surrounding single-stranded DNA for precise and rapid nucleic acid detection.^162,163^ Emerging studies suggest that CRISPR-based systems can also be adapted to detect a broader range of targets, including proteins, antibiotics, metal ions, and amino acid metabolites. However, CRISPR-based detection is currently slightly less sensitive than the widely used RT-PCR test.

For example, scientists reported that CRISPR-Cas-based diagnostics offers a safe, non-invasive way to detect conditions like Trisomy 13, 18, and 21 using the cell-free fetal DNA (cffDNA) just extracted from the maternal blood sample during the early gestation period using the kit method. In this process fetal DNA is gently extracted and amplified with fluorescent primers and targeted by specific guide RNAs and Cas enzymes, then detection of 3 targets will be favored by adding reporters for Cas12f, Cas12b, and Cas12a in HEX (hexachlorofluorescein), TEX (TEX615), and FAM channels to help with early detection of any chromosomal issues with high sensitivity and specificity. Then, a clear fluorescent signal generated from the DNA-Cas-sgRNA complex confirms the presence of trisomy. It allows quick, affordable testing and avoids the risks of traditional procedures like amniocentesis.^164^

A rapid diagnosis of SARS-CoV-2 is essential to enable timely treatment for COVID-19; reverse transcription-polymerase chain reaction (RT-qPCR) assays were developed to detect the novel coronavirus within 4–6 hours and are considered the gold standard for identifying viral nucleic acids due to their high sensitivity and specificity.^165^ However, these assays require expensive equipment and trained personnel, hindering their accessibility in resource-constrained settings. On the other hand, serology tests are faster and require minimal equipment but are less effective for diagnosing acute SARS-CoV-2 infections, as antibody responses can take several days to weeks to develop. To address the need for instrument-free nucleic acid detection, various isothermal amplification methods—such as Recombinase Polymerase Amplification (RPA) and Loop-mediated Isothermal Amplification (LAMP)—have been developed. These techniques are simpler, faster, and more cost-effective than conventional PCR assays.^166^ Alternative approaches based on combinations of RPA, LAMP and CRISPR-mediated detection have been developed. These methods enable instrument-free, sensitive, precise and rapid point-of-care testing (POC) for COVID-19.^167^ In this process, patient samples (usually nasal or throat swabs) are collected and processed to extract viral RNA, which is then amplified using isothermal techniques like RT-LAMP or RPA. CRISPR enzymes (*e.g.*, Cas12 or Cas13), guided by virus-specific RNA sequences, bind the amplified target and activate *trans*-cleavage, cutting reporter molecules to produce a detectable fluorescent or colorimetric signal.

#### 3.1.1 SHERLOCK

The Specific High Sensitivity Enzymatic Reporter UnLOCKing (SHERLOCK) diagnostic technique is a two-step reaction assay that combines RPA pre-amplification (T7 RNA polymerase) with a CRISPR-Cas13a enzyme complex and fluorescence reporter for ultra-sensitive, specific, and rapid detection of nucleic acid.^45,168^ With a setup time of under 15 minutes, this system provides fluorescence and colorimetric readouts, delivering results in under an hour for the rapid and sensitive detection of diseases such as COVID-19, Zika, and cancer biomarkers. SHERLOCK reduces dependence on RT-qPCR equipment, but it also presents some challenges including time-consumption, complexity, potential cross-contamination, difficulties in quantification and multiple fluid handling steps, complicating its deployment outside clinical labs.^169^

#### 3.1.2 STOP-covid

The SHERLOCK testing in one pot for COVID-19 (STOP-Covid) is a one-step reaction assay that combines CRISPR-Cas12b (AapCas12b-crRNA) with RT-LAMP and LAMP amplification primers in isothermal amplification buffer,^170^ developed as an alternative to the RPA method. As a rapid, point-of-care diagnostic tool it is capable of detecting SARS-CoV-2 with results available in under an hour – approximately 40 minutes using a fluorescence or 70 minutes with a lateral flow readout. It eliminates the need for RNA extraction, operates at a single temperature, and requires only one fluid handling step. This simplified test that provides sensitivity comparable to RT-qPCR based SARS-CoV-2 tests has some limitations like it can detect 100 copies of viral genome per reaction from saliva or nasopharyngeal swabs.^46^ To enhance accessibility, it is recommended that the assay should be further developed into a low-cost format with a modular heater to streamline the workflow and facilitate broader point-of-care use.

#### 3.1.3 DETECTR

DNA Endonuclease Targeted CRISPR Trans Reporter (DETECTR) is a visual, faster, low-cost, accurate and one-pot reaction assay, in which CRISPR-Cas12a (Cas12-crRNA) in combination with RT-LAMP and LAMP detects dsDNA targets.^171^ Upon binding to its target, Cas12 cleaves nearby single-stranded DNA (ssDNA) probes, generating a fluorescence signal. The assay requires only 30–40 min to detect viral genes including the E-gene and N-gene of the virus, as well as the human RNase P-gene, which serves as an internal controller with a detection limit of approximately 10 copies/µL viral nucleic acid. It has been applied for the rapid detection of Human Papillomavirus (HPV), coronavirus and other DNA-based diseases. In the case of respiratory swab samples for coronavirus detection, the system demonstrated 90% sensitivity and 100% specificity, without the need for expensive laboratory equipment.

#### 3.1.4 HOLMES

**Fig. 4:**
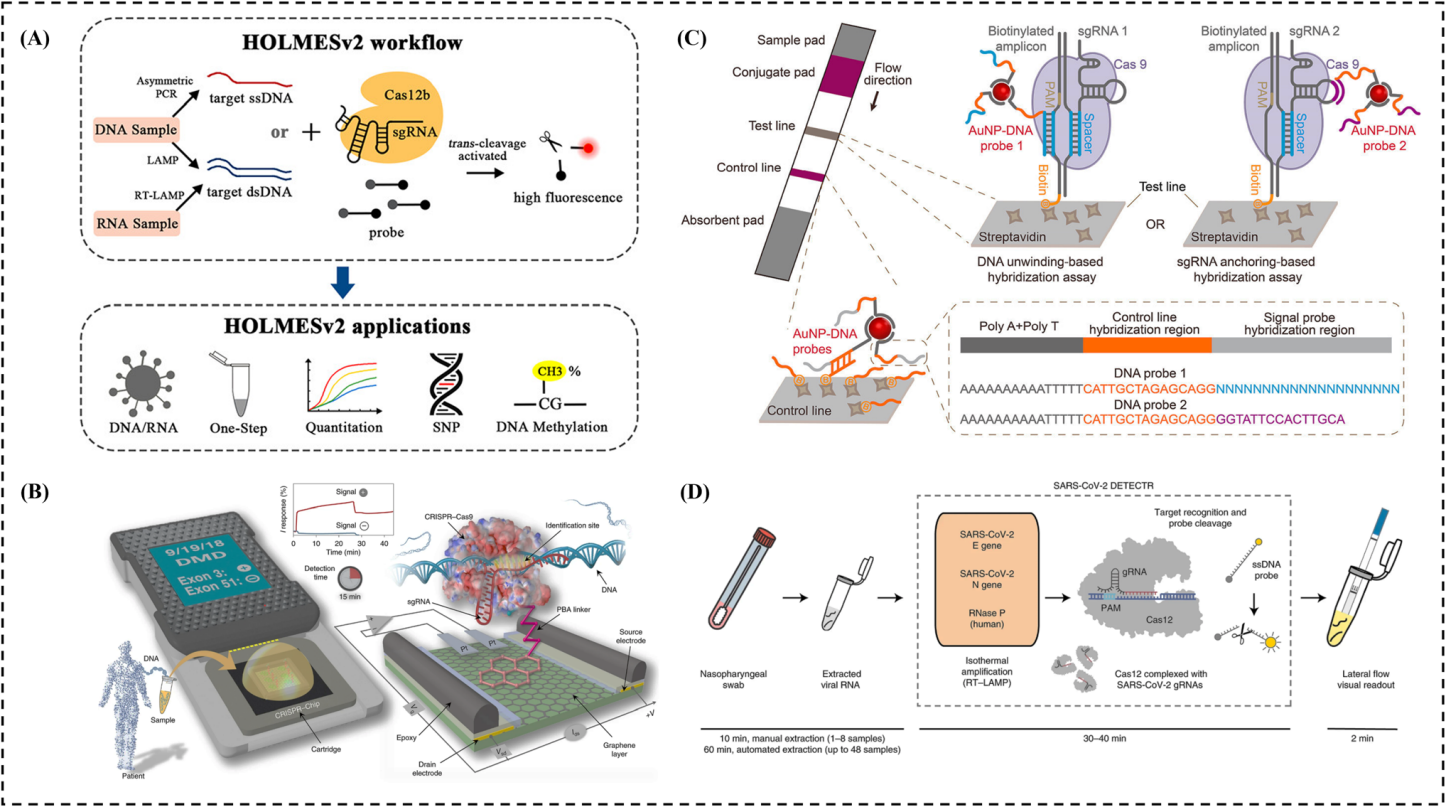
CRISPR-based diagnosis system: (a) detection using the HOLMESv2 technique, figure adapted from ref. 172. Copyright^©^ 2020, American Chemical Society. (b) dCas9-based electrochemical detection using a handheld device, figure adapted from ref. 173. Copyright^©^ 2019, Reza Hajian *et al.* (c) detection using the CASLFA technique, figure adapted from ref. 174. Copyright^©^ 2020, American Chemical Society. (d) Detection using the AIOD-CRISPR technique, figure adapted from ref. 47. Copyright^©^ 2020, James P. Broughton *et al.*

The one-hour low-cost multipurpose highly efficient system (HOLMES) uses Cas12 or Cas13 to detect DNA and RNA.^122^ It delivers results in less than an hour making it useful for infectious disease diagnostics and genetic testing. To develop HOLMESv2, Linxian *et al.* utilized Cas12b (formerly known as C2c1), a thermophilic RNA-guided endonuclease from the type V-B CRISPR-Cas system^172^ (Fig. 4A). This version can discriminate single nucleotide polymorphisms (SNPs), detect RNA viruses, human cellular mRNA, and circular RNA, as well as quantify target DNA and assess its methylation status in various human cell lines. Additionally, the detection of target nucleic acids and their amplification were integrated into a single system to streamline the workflow and minimize the risk of cross-contamination.

#### 3.1.5 CRISPR-chip

The CRISPR-Chip is a label-free biosensor for nucleic acid testing, with output signals that can be measured using a basic handheld reader. It utilizes the gene-targeting capability of catalytically deactivated CRISPR-associated protein 9 (Cas9), which is complexed with a specific single-guide RNA and immobilized on a transistor. The basic idea is to integrate graphene-based electrical transistors with dCas9 by connecting to a specific DNA molecule; the dCas9/gRNA complex can alter the conductivity of the transistor and generate a detecting signal. Target DNA at concentrations as low as 7 fM can be detected in 15 minutes by the very sensitive graphene transistor utilized in CRISPR circuits. However, direct detection without DNA amplification can result in higher limits of detection (*i.e.* lower sensitivity), especially for low-copy samples, and requires complex fabrication involving precise nanofabrication. For example, Hajian R. *et al.* developed the CRISPR-chip to analyze DNA samples from HEK293T cell lines expressing blue fluorescent protein, as well as clinical DNA samples (Fig. 4B).^173^ Exon mutations in clinical samples from patients with Duchenne muscular dystrophy were identified using this portable, amplification-free technique.

#### 3.1.6 CASLFA

CRISPR associated lateral flow assay (CASLFA) is a portable, rapid, highly sensitive, specific, cost effective, user friendly diagnostic method.^174^ The system integrates PCR-based or RPA-based DNA/RNA amplification with a CRISPR/Cas9 lateral flow nucleic acid detection assay.^174^ To target specific nucleic acid sequences, the method uses biotinylated 5′ primers during PCR or RPA. The CASLFA product includes the CRISPR/Cas9 target sequence, a hairpin guide RNA (gRNA), a biotin tag, and gold nanoparticle (AuNP)-DNA probes 1 and 2. The CRISPR/Cas9-gRNA complex is directed by the gRNA to bind the target sequence. It interacts with the biotin-labeled AuNP-DNA probes and streptavidin, enabling visual detection of the specific DNA sequence on the lateral flow strip, but the lateral flow formats typically only support 1–2 targets per strip, which restricts broad pathogen or mutation screening and opening tubes post-amplification increases the risk of aerosol contamination, which can lead to false positives. For example, Wang, X. *et al.* demonstrated CASLFA's ability for quick detection of Listeria monocytogenes, GMOs, and ASFV with high specificity within one hour even at low genome levels. It identified 27 ASFV-positive cases from 110 serum samples, when tested outside the lab matching RT-PCR results with 100% accuracy. With its speed, accuracy, and minimal equipment needs, CASLFA is a promising tool for on-the-spot diagnostics, especially in low-resource settings (Fig. 4C).^174^ It interacts with the biotin-labeled AuNP-DNA probes and streptavidin, enabling visual detection of the specific DNA sequence on the lateral flow strip, but the lateral flow formats typically can only support 1–2 targets per strip, which restricts broad pathogen or mutation screening and opening tubes post-amplification increases risk of aerosol contamination, which can lead to false positives.

#### 3.1.7 AIOD-CRISPR

All-In-One dual CRISPR systems (AIOD-CRISPR) were developed by combining RPA pre-amplification with Cas12a (a pair of crRNAs introduced to initiate dual CRISPR-Cas12a detection and improve detection sensitivity) for detection and enhancement of sensitivity. This system enables simple, rapid, one-pot, visual, ultrasensitive, and highly specific detection of nucleic acids. AIOD-CRISPR exhibits high specificity in detecting the N gene of SARS-CoV-2 using the human RPP30 gene as a control, while minimizing cross-reactions with non-SARS-CoV-2 targets as a limitation. For example, Ding, X. *et al.* developed the AIOD-CRISPR assay—a simple, highly sensitive, low-cost heat source, one-pot test using dual CRISPR-Cas12a to help with early detection of COVID-19, which delivers clear results in 20 minutes, with even tiny amounts of the virus's genetic material, and slow the spread of SARS-CoV-2. It targets the nucleoprotein gene without needing a PAM site giving results that match RT-PCR accuracy using clinical swab samples (Fig. 4D).^47^

### 3.2 CRISPR-based real-time tracking and visualization

Real-time tracking is used to monitor the chromosome dynamics during genome editing. Traditional methods for real-time monitoring have several limitations, including low temporal resolution, destructive sampling techniques, limited sensitivity, and inadequate spatial resolution. Real-time visualization provides a deeper understanding of CRISPR-Cas9 interactions with DNA, its editing efficiency, and potential off-target effects, all of which are crucial for optimizing CRISPR-based genome editing and ensuring its safety in therapeutic applications. Consequently, real-time tracking of CRISPR-mediated genome editing has emerged as a key research goal. In today's world, various CRISPR-based bioimaging techniques are available.^175^

Visualization of chromosome dynamics is crucial for understanding various fundamental intra-nuclear processes. For example, Shaho *et al.* developed an innovative method utilizing a modified single-guide RNA (sgRNA) within the CRISPR/Cas9 system to enable dual-color chromosomal locus imaging.^176^ This approach involves structure-guided engineering of the sgRNA by inserting RNA aptamers that specifically bind to fluorescent protein-tagged effectors, thereby optimizing the imaging strategy. Moreover, studies have demonstrated that this sgRNA-based labeling technique exhibits significantly greater resistance to photobleaching compared to Cas9-based labeling, due to the rapid exchange dynamics of the RNA aptamer-binding effectors.

Another method was established by Yang J. *et al.*, employing aptamer-modified DNA origami as a delivery vehicle to dynamically track target loci in a human breast cancer cell line (MCF-7).^177^ The ribonucleoprotein (RNP) complex, consisting of dCas9-EGFP and cyanine-5 (Cy5)-labelled gRNA, is efficiently encapsulated, protected, and delivered by a barrel-shaped, hollow DNA origami nanostructure known as TUBE. Following a 6 hour incubation with the nanostructures, high-resolution multi-color and labelling of target genes in living cells is achieved without affecting cell viability, proliferation, or chromosome dynamics, demonstrating the rapid and biocompatible nature of this DNA origami-based delivery method.

Furthermore, to investigate the spatiotemporal dynamics and functional role of non-repetitive genes in living cells, Chen B *et al.* developed a CRISPR-Tag system that utilizes 1 to 4 highly active sgRNAs to specifically label protein-coding genes with a high signal-to-noise ratio for real-time imaging *via* wide-field fluorescence microscopy.^178^ Incorporating 2–6 CRISPR-targetable DNA sequence repeats from *Caenorhabditis elegans* into human genes helps to efficiently label endogenous genes like HIST2H2BE, LMNA, and HSPA8 with fluorescent dCas9. Robust imaging of repetitive sequences in telomeres and coding genes in living cells has also been demonstrated using a structurally optimized single-guide RNA (sgRNA) in combination with an EGFP-tagged, endonuclease-deficient Cas9 (dCas9) protein.

**Fig. 5:**
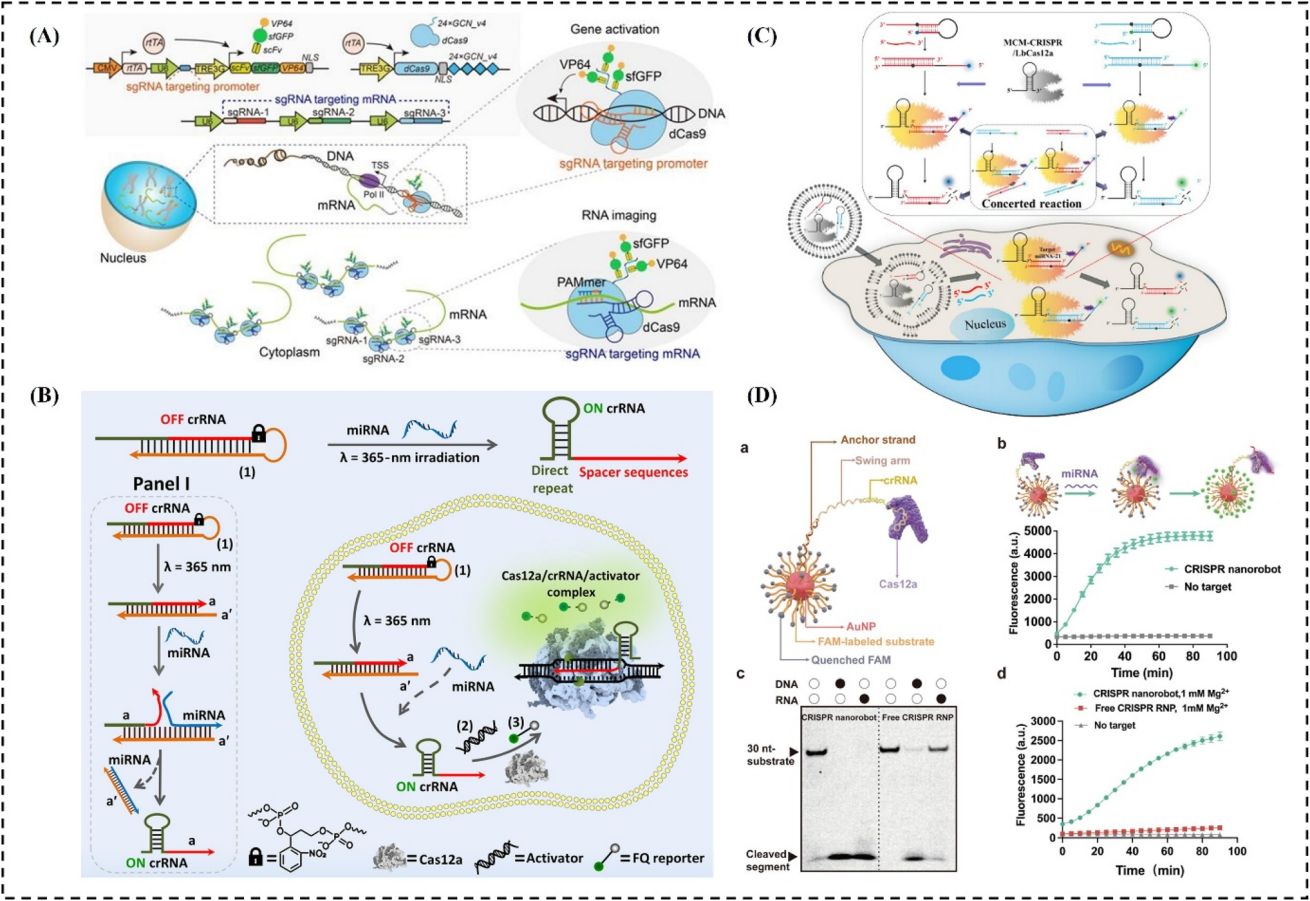
CRISPR-based bioimaging strategies. (A) Schematic diagram of the SunTag-mediated single-molecule RNA imaging method (CRISPR-Sunspot), figure adapted from ref. 179 Copyright^©^ 2020, Sun *et al.* (B) diagram illustrating how a response module linked to the CRISPR-Cas12a (LAC12a) system can be light-activated to release cellular protection and enhance miRNA detection, figure adapted from ref. 180 Copyright^©^ 2024, the American Association for the Advancement of Science. (C) Diagram of an MCM-CRISPR/Cas12a system for sensitive and specific imaging of multiple microRNAs within cells, figure adapted from ref. 179 Copyright^©^ 2024, Chen X. *et al.* (D) schematic illustration of the construction and application of CRISPR nanorobots consisting of an AuNP functionalized with molecular constructs to enable CRISPR activity, figure adapted from ref. 181 Copyright^©^ 2024 American Chemical Society.

Sun *et al.* developed a CRISPR-Sunspot imaging system based on SunTag technology to enhance signals from single-molecule mRNA in living cells.^179^ Using CRISPR-Sunspot, the co-localization of Camk2a mRNA with the regulatory protein Xlr3b in neurons was successfully monitored (Fig. 5A). Liu H. *et al.* assembled a light-activated CRISPR-Cas12a (LAC12a) system for enhanced detection of miRNA-21 and its imaging in living cells (Fig. 5B).^180^ This study is significant as it introduces a photoactivated CRISPR-Cas12a platform for improved miRNA detection, leveraging spatiotemporal imaging capabilities that enable single-cell resolution analysis of miRNA expression.

Furthermore, Chen X. *et al.* developed a mini crRNA-mediated CRISPR/Cas12a (MCM-CRISPR/Cas12a) system for sensitive and targeted imaging of miRNA-21, miRNA-155, and miRNA-10b both inside and outside cells (Fig. 5C).^182^ Their results showed that molecular beacons (MBs) in the MCM-CRISPR/Cas12a system produced significantly higher fluorescence intensity compared to MBs opened directly by the target microRNAs. Yuan A. *et al.* designed a study to overcome the limitations of traditional methods that require supplying high concentrations of substrates into cells.^181^ In their approach, high local concentrations of both enzyme and substrate were achieved within a nanorobot, in which crRNA-Cas12a RNP complexes hybridize with thousands of anchor strands and hundreds of substrate strands conjugated to an AuNP scaffold, forming the functional nanorobot (Fig. 5D).

### 3.3 Medical healthcare (cancer therapy and gene editing)

Gene therapy represents a promising therapeutic approach that involves the modification or replacement of defective genes to correct genetic mutations, thereby offering the potential for curative treatment or prevention for various human genetic disorders caused by gene mutations including point mutations, gene defects or abnormal gene expression.^183^ This approach holds therapeutic potential for conditions such as cancers (*e.g.*, tumors), monogenic diseases (*e.g.*, sickle cell disease, β-thalassemia), hematological malignancies like leukemia, and viral infections (*e.g.*, hepatitis B virus-induced hepatocellular carcinoma, human papillomavirus-associated cervical cancer, and HIV-induced acquired immunodeficiency).^184^ Unlike conventional treatment modalities, gene therapy represents a transformative paradigm that targets the underlying genetic etiology, offering the possibility of long-term or permanent cures. The CRISPR-Cas9 genome editing system has emerged as a transformative tool in this domain, owing to its precision, efficiency, and programmability. It has been extensively employed in both preclinical and clinical investigations for therapeutic gene correction.

Conventional cancer treatments such as surgery, radiation and chemotherapy can slow down cancer progression and extend patient survival. However, these methods often come with the risk of the cancer coming back or the body becoming resistant to the drugs, that can result in a poor outlook for patients.^185^ Moreover, the lack of precision in chemotherapy and radiation therapy can cause severe side effects, and can sometimes even be life-threatening. Recently, the US Food and Drug Administration (FDA) approved CAR-T cell therapy for cancer treatment.^186^ CAR-T cell therapy is a form of immunotherapy that involves modification of T cells (engineered T cells) to express chimeric antigen receptors, known as CAR T cells.^187^ CARs are specially engineered proteins that help T cells recognize and attack cancer. They are created by linking single-chain variable fragments to transmembrane and intracellular signaling regions, often including one or two co-stimulatory domains. These recombinant antigen receptors can reprogram the specificity and function of T lymphocytes, are often enhanced with additional support signals, and enable a stronger, more targeted anti-tumor response.^188^

However, there is a continued strong need for innovative cancer therapies, and CRISPR/Cas9 technology holds the potential to significantly transform cancer treatment and could be a game-changer in the way the disease is treated.^189^ For example, Stadtmauer *et al.* reported a first-in-human phase 1 clinical trial (clinicaltrials.gov (http://clinicaltrials.gov); trial NCT03399448) where three genes (TRAC, TRBC, and PDCD1) of T cells extracted from the body of three patients, two of them with advanced treatment-resistant myeloma and one with metastatic sarcoma, were targeted and disrupted by the multiplex CRISPR-Cas9 gene editing tool to boost the cells' ability to fight tumors.^190^ Here, the edits removed endogenous TCRs and PD-1 to enhance T cell function and persistence, while reducing immune exhaustion, and a cancer-specific TCR transgene (NY-ESO-1) was added to redirect T cell specificity toward tumor antigens. Then modified T cells were safely infused back into the patient's body and showed stable engraftment for ≥9 months. As a result, all patients tolerated the therapy well, with no serious side effects linked to the gene edits. The TCR α chain gene (TRAC) showed the most effective editing, and reductions in NY-ESO-1/LAGE-1 antigens were observed in myeloma patients. While travelling to tumor sites, the cells showed a drop in key cancer markers. Off-target effects were rare, and chromosomal translocations occurred *in vitro* but declined post-infusion. The study confirms that CRISPR-Cas9 editing is clinically feasible with potential for advancing cancer immunotherapy.

Furthermore, CRISPR-Cas9 facilitates the generation of genetically engineered animal models, enabling in-depth studies of disease mechanisms and the development of targeted interventions.^191^ For example, Oura S. *et al.* reported an engineered variant of the *Streptococcus pyogenes* Cas9 nuclease, termed SpCas9-NG, which recognizes a relaxed NGN protospacer adjacent motif (PAM), thereby significantly expanding the range of targetable genomic loci. In their study, they demonstrated the potential of SpCas9-NG-mediated genome editing to correct the pathologically expanded CAG repeat tract characteristic of Huntington's disease (HD). By specifically targeting the boundary of the CAG repeat expansion in embryonic stem (ES) cells derived from an HD mouse model, SpCas9-NG facilitated precise genome editing, leading to a precise contraction of the aberrant repeat region.^192^

**Fig. 6:**
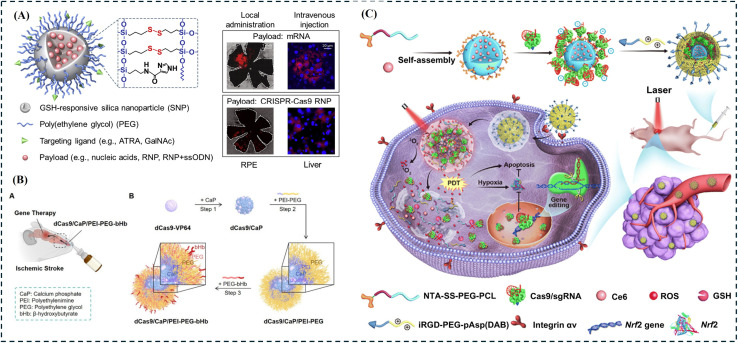
Applications of CRISPR-based technologies in gene editing. (A) Schematic illustration of a stimulus-responsive silica nanoparticle (SNP) for intracellular trafficking of CRISPR genome editors (*e.g.*, RNP and RNP + ssODN) and nucleic acids (*e.g.*, DNA and mRNA), figure adapted with permission from ref. 193 Copyright^©^ 2021 Elsevier. (B) Schematic representation of dCas9/CaP/PEI-PEG-bHb administered intranasally for the treatment of ischemic stroke, figure adapted from ref. 194 Copyright^©^ 2024 Jee-Yeon Ryu *et al.* (C) schematic diagram of NIR-sensitive and reducing agent-sensitive nitrilotriacetic acid-decorated micellar nanoparticles encapsulated with anticancer photosensitizer Ce6 and Nrf2-targeting Cas9/sgRNA for application in photodynamic therapy, figure adapted from ref. 195 Copyright^©^ 2020 Deng S. *et al.* American Association for the Advancement of Science.

Morever, Wang Y. *et al.* reported a stimulus-responsive silica nanoparticle (SNP, ∼50 nm) capable of encapsulating biomacromolecules with high loading efficiency and releasing cargo in response to glutathione (GSH) *via* disulfide crosslinking (Fig. 6A).^193^ These SNPs demonstrated efficient and biocompatible delivery of nucleic acids (DNA, mRNA) and CRISPR genome editors (Cas9/sgRNA RNPs), enabling effective genome editing. *In vivo* studies further showed successful mRNA and RNP delivery to murine retinal pigment epithelium (RPE) and liver cells, achieving targeted genome editing. In another study, nanoparticles containing a protein-based CRISPR/dCas9 system targeting Sirt1 were prepared, and their intranasal delivery was evaluated in a mouse model of ischemic stroke (Fig. 6B).^194^ Intranasal administration of this system (dCas9/CaP/PEI-PEG-bHb nanoparticles) improved survival following permanent middle cerebral artery occlusion and reduced cerebral edema *via* the upregulation of target gene Sirt1 in the brain.

Deng S. *et al.* designed near-infrared (NIR)- and reducing agent-responsive nanoparticles and achieved controlled release of CRISPR-Cas9 RNPs along with the anticancer photosensitizer chlorin e6 (Ce6) in a mouse animal model (Fig. 6C).^195^ They validated the synergistic effects of Nrf2 gene editing and Ce6-mediated photodynamic therapy *in vivo*. These approaches demonstrate the potential of CRISPR-based gene-editing strategies, synergistically integrated with nanotechnology and anticancer therapeutics, for advanced biomedical applications.

### 3.4 Agricultural and environmental applications

Food security remains a critical challenge as the global population grows, with agricultural productivity needing to increase sustainably to tackle malnutrition and hunger. Projections indicate that global food demand will rise by approximately 100–110% between 2005 and 2050, potentially exacerbating environmental impacts depending on the methods used for agricultural expansion.^196^ Focusing on efficient farming practices and improved technologies could help meet this demand while minimizing land clearing and reducing greenhouse gas emissions and environmental harm.

The CRISPR-Cas9 site-specific genome editing technology is a promising approach towards global food security/safety, crop improvement and livestock engineering.^197^ As a rapid, efficient and cost-effective technology, CRISPR-Cas9 enables the development of genetically modified organisms (GMOs) with enhanced traits. This approach facilitates the biofortification of major crops with essential vitamins and minerals, thereby improving their nutritional value.^198^ Furthermore, CRISPR has been successfully employed to enhance crop resilience against various abiotic stresses such as drought, salinity and heat,^199^ as well as biotic stresses including disease resistance.^200^ The adoption of next-generation CRISPR platforms—including methods like *de novo* domestication of plants from scratch, editing genes in specific tissues, using prime editing for greater accuracy and fine-tuning of gene expression within multidisciplinary breeding strategies—holds significant promise for the development of climate-resilient crops, ultimately contributing to sustainable global food security.^201,202^

CRISPR-Cas technology has emerged as a powerful tool for crop quality enhancement, with a primary focus on improving physical appearance—such as shape, size, color and texture for prolonged shelf life.^203–205^ Additionally, this technology has been employed to enhance nutritional quality by increasing carotenoid content and GABA levels, modifying fatty acid composition, biofortifying micronutrients and eliminating anti-nutritional factors.^206–208^ CRISPR has also contributed to enhancing edible quality and palatability, such as improving cooking and eating quality and optimizing flavor, thus facilitating the development of crop varieties with preferred consumer traits.^209–211^

The CRISPR/Cas toolkit has expanded into the RNA-editing domain with the advent of Cas13—a versatile “Swiss Army knife” for plant researchers.^212^ This RNA-targeting system offers unprecedented opportunities to manipulate plant transcriptomes. Nuclease-dead versions of Cas13 enable real-time visualization of RNA dynamics in living cells and facilitate precise, programmable RNA editing through base-specific deamination or chemical conjugation.^213,214^ Cas13 offers several advantages over traditional technologies, including scalability, multiplex RNA targeting, and the ability to target non-coding nuclear transcripts and pre-mRNA when fused with nuclear localization signals.

While CRISPR/Cas9 has made significant developments in plant genome editing, its application in plant pathogen diagnostics remains underdeveloped. Early pathogen detection could help farmers to manage diseases and reduce crop losses. CRISPR-Cas9 enables quicker and more efficient development of disease-resistant crops, offering an alternative to traditional breeding methods which are slow and labor-intensive.^167^For example, the development of DNA detection systems using Cas12a, in conjunction with highly active crRNAs and lateral flow assays (LFAs), has shown promise for low-cost, efficient, and field-deployable diagnostics in plant disease surveillance and GMO monitoring.^215^ Moreover, recent studies showed that CRISPR-Cas9-mediated inactivation of the Downy Mildew Resistance 6 (DMR6) gene in *Arabidopsis thaliana* confers resistance to multiple pathogens. Similarly, targeted mutagenesis of the DMR6 ortholog (*SlDMR6-1* orthologue Solyc03g080190) in tomato also leads to broad-spectrum disease resistance against *Pseudomonas syringae*, *P. capsica*, *Xanthomonas* spp, *pv. tomato* and *Phytophthora capsica* as this gene is significantly upregulated upon infection.^216^

**Fig. 7:**
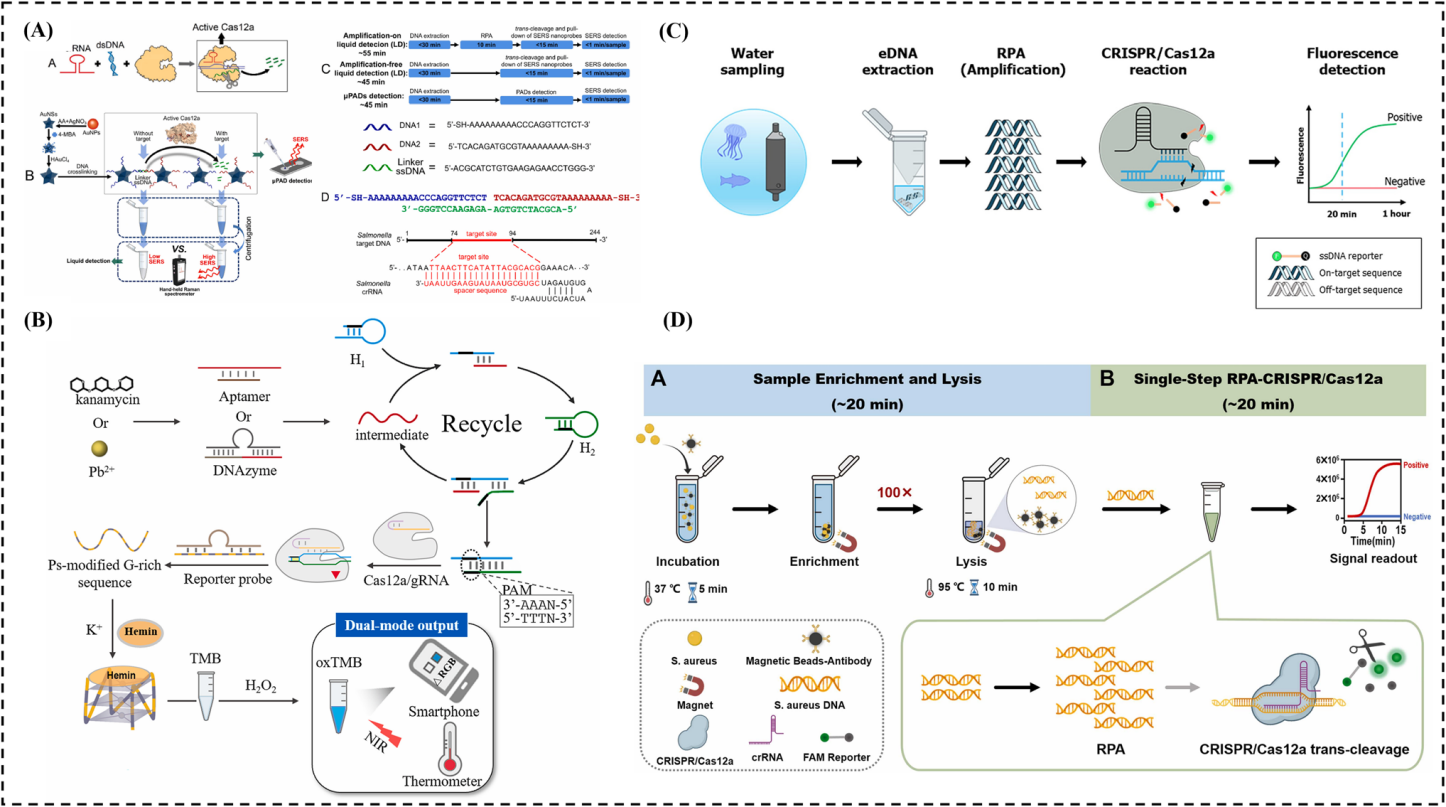
Applications of CRISPR/Cas9 in agriculture and environmental sustainability. (A) Schematic design of the proposed CRISPR-Cas12a-based biosensor employing gold nanostar@4-mercaptobenzoic acid@gold nanoshell structures (AuNS@4-MBA@Au) for SERS-based bacterial detection, figure adapted with permission from ref. 217 Copyright^©^ 2022 Elsevier. (B) Schematic illustration of a ‘signal-on’ colorimetric/photothermal biosensor (psG4-CHA/Cas) developed for portable detection of environmental contaminants, utilizing a catalytic hairpin assembly (CHA) with Cas12a for amplification and phosphorothioate-modified G4 as a reporter, figure adapted with permission from ref. 219 Copyright^©^ 2024 Elsevier. (C) Schematic diagram of the RPA-CRISPR/Cas12a environmental DNA (eDNA) assay, figure adapted with permission from ref. 220 Copyright^©^ 2024 Elsevier. (D) Schematic illustration of the RPA-CRISPR/Cas12a-based platform for *S. aureus* detection, figure adapted with permission from ref. 218 Copyright^©^ 2024 Elsevier.

The emergence of infectious diseases caused by bacterial pathogens, including foodborne species such as *Staphylococcus aureus* (*S. aureus*), represents a persistent global public health concern due to their serious microbiological risks and potential to cause food poisoning and other infectious diseases. Several studies have been reported to develop rapid, efficient, and ultrasensitive methods for detecting *S. aureus* in food samples. For example, Zhuang J. *et al.* developed a microfluidic paper-based analytical device (µPAD) integrating recombinase polymerase amplification (RPA) with CRISPR/Cas12a (RPA-Cas12a-µPAD), for ultrasensitive surface-enhanced Raman scattering (SERS) detection^217^ (Fig. 7A). The platform achieved a dynamic detection range of 1–10^8^ CFU mL^−1^ with a limit of detection of approximately 3–4 CFU mL^−1^ for *S. typhi* in spiked milk and meat samples. Precise detection of foodborne pathogens in dietary samples was accomplished within 45 minutes using the RPA-Cas12a-µPAD. In another study, Yujie Ma *et al.* developed a magnetic enrichment cascade single-step recombinase polymerase amplification (RPA)-CRISPR/Cas12a assay (Fig. 7D).^218^ This assay could detect as low as 10 CFU mL^−1^ of *S. aureus* and 1 copy per µL (five copies per reaction) of extracted DNA within 40 minutes. Moreover, the assay produced results consistent with qPCR and successfully detected *S. aureus* in real food samples, including lettuce, pork, orange juice, and lake water.

CRISPR technology has revolutionized molecular biology with broad applications in environmental biotechnology.^219,221^ It enables the engineering of microorganisms for bioremediation, degradation of pollutants, and clean-up of oil spills and heavy metal contamination.^220^ For example, Kai Shi *et al.* designed a “signal-on” colorimetric/photothermal biosensor (psG4-CHA/Cas) that combines catalytic hairpin assembly (CHA) with Cas12a as an amplification strategy and phosphorothioate-modified G4 as a reporter for the portable detection of environmental contaminants (Fig. 7B).^219^ For the colorimetric detection of Pb^2+^ and kanamycin, a smartphone was employed. The detection limits for kanamycin and Pb^2+^ were found to be 100 pM and 50 pM, respectively. Additionally, a portable thermometer was used, achieving detection limits of 10 pM for kanamycin and 8 pM for Pb^2+^.

CRISPR supports the development of fourth-generation biofuels, control of invasive species, genetic enhancement for ecosystem restoration, and modulation of methane emissions.^221^ Environmental DNA (eDNA) research enables near real-time global species monitoring, offering enormous potential to advance biodiversity science and conservation efforts. For example, Kim K. *et al.* developed a two-step RPA-CRISPR-Cas12a eDNA assay, comprising target eDNA amplification followed by a CRISPR-Cas12 reaction, for the detection of seasonal *Chrysaora pacifica* (Fig. 7C).^220^ The assay results were validated and compared using visual observations of jellyfish, absolute quantification *via* dPCR, and eDNA metabarcoding. The RPA-CRISPR-Cas12a assay, with its isothermal operation, high specificity, sensitivity, and precision, demonstrates significant potential for low-cost, on-site eDNA detection of non-indigenous species. Additionally, CRISPR/Cas-based biosensors offer promising tools for monitoring environmental health, while CRISPR diagnostics enable early detection of pathogens and contaminants, such as waterborne microbes.^222,223^ Furthermore, by integrating with nanotechnology it forms smart nanostructure hybrids that can enhance environmental clean-up and long-term ecological sustainability.^224^

#### 3.4.1 De-extinction special (resurrection of extinct species)

When Jurassic Park premiered in the 1990s, the idea of bringing dinosaurs back through genetic engineering seemed like pure science fiction. However, public fascination with de-extinction predates even that. In 1984, the idea of creating a woolly mammoth-elephant hybrid captured national attention, with over 350 newspapers across the U.S. covering story.^225^ The excitement was sparked by an article in the April issue of *MIT Technology Review*, which introduced a fictional creature called the “montelephas”. It turned out to be an April Fool's prank, but the massive media response showed just how believable and captivating the idea really was. While the original story was a hoax, it revealed deep public interest in the idea of reviving extinct species. Today, this concept is shifting from fantasy to scientific possibility. Advances in biotechnology are making the revival of recently extinct species—such as the gastric brooding frog, woolly mammoth, Tasmanian tiger, and passenger pigeon—more plausible than ever. Scientists are exploring multiple approaches, including back-breeding, cloning, and gene editing, to reconstruct lost species even when original DNA is poorly preserved. Among these tools, CRISPR gene editing stands out for its precision and efficiency in enabling de-extinction efforts.^226^

By inserting carefully edited DNA sequences—representing traits of an extinct species—into the genome of a closely related living species, researchers can generate hybrid organisms. These hybrids are not exact replicas of the extinct species, but rather new organisms that retain essential features of the original ones while incorporating traits from their modern relatives. According to Colossal Biosciences, a Dallas–Texas based bioscience and genetic engineering company focused on species de-extinction,^227^ they have achieved a groundbreaking milestone: the successful birth of three healthy dire wolves, a species that roamed North America but has been extinct for over 12 500 years.^228^ While many dire wolf fossils were discovered in the La Brea Tar Pits in Los Angeles, the tar didn't preserve species DNA.

Reintroducing revived species (mainly the keystone species) into environments where they can thrive not only adds to biodiversity but also strengthens the resilience of entire ecosystems.^229^ Colossal Biosciences' pioneering conservation efforts exemplify how advanced genetics can be harnessed not merely to preserve endangered species, but to revive ecosystems. By introducing woolly mammoth traits into Asian elephants, the goal extends beyond species conservation to the restoration of the ancient Arctic grasslands once inhabited by mammoths. Releasing mammoth-elephant hybrids into the tundra could stimulate large-scale ecological recovery. Their movement and behaviour, such as trampling permafrost, toppling low-oxygen trees, and creating open spaces for carbon-sequestering grasses, could help rejuvenate the tundra ecosystem. This transformation has the potential to enhance carbon capture, mitigate greenhouse gas emissions, and contribute meaningfully to global climate regulation.

## 4 Limitations of CRISPR-Cas9 technology

The development of the CRISPR-Cas9 system has provided transformative potential and widespread applications across many fields, including medical therapeutics, agricultural engineering, and biological research,^230,231^ as it offers relatively simple, precise, programmable, and versatile tools and an efficient method for altering DNA sequences in living organisms. However, despite their promise, the efficacy of first-generation CRISPR-based gene editing tools is often limited by several challenges such as efficiency of delivery, off-target effects, targeting scope, half-life of CRISPR components, and endogenous DSB repair mechanism efficiency to achieve genomic edits, ethical concerns and the need for real-time monitoring during editing processes at the cellular level. This will enable a deeper understanding of how CRISPR-Cas9 interacts with DNA, which is critical for optimizing the CRISPR editing system and ensuring its safety in therapeutic applications.^88^

Hsu *et al.* reported that decreasing the content of Cas9 protein in the CRISPR-Cas9 system will reduce or overcome the off-target effect challenges, but it will also cause low efficiency of targeted cutting, which is the main role of CRISPR technology.^232^ Off-target effects (OTEs) occur when CRISPR-Cas9 binds to and cuts DNA even when mismatches exist between the guide RNA and the target sequence. Although this mechanism helps defend against viruses in nature, in genome editing it can lead to unintended modifications at similar genomic sites, potentially causing mutations or chromosomal alterations. Therefore, scientists are working to improve the accuracy and safety of CRISPR for medical applications, with ongoing efforts focused on enhancing detection techniques such as *in silico* tools and high-throughput sequencing.^233^

Other researchers also designed different techniques to resolve these challenges by continuous enhancement or upgradation of the specificity by improvement of guide RNA (gRNA) design, bioinformatic tools and high-fidelity Cas9 enzymes for recognition of sites as well as a series of structural modifications to the Cas9 protein. The modifications of Cas9 protein generally result in prime editing, base editing, transcriptional modulation (gene regulation) and RNA editing (Fig. 1(c)). For example, in 2020, Lin Q. *et al.* reported prime editing created by fusing a CRISPR-Cas9 nickase (H840A) with reverse transcriptase and guided by pegRNAs to make precise changes in DNA without needing donor templates or causing double-strand breaks. This system was tailored in plants by optimizing codons, promoters, and editing conditions, which successfully produced point mutations, insertions, and deletions in rice and wheat protoplast. In rice, prime-edited plants were regenerated with editing efficiencies reaching up to 21.8%.^234^ Newby G.A. *et al.* (2021) demonstrated an alternative approach for modifying Cas proteins to treat sickle cell disease, which is caused by a mutation in the HBB gene. Using the specialized adenine base editor ABE8e-NRCH, they converted the harmful SCD variant HBBS gene to the harmless HBBG (Makassar β-globin) by introducing the editor and guide RNA into blood stem cells from SCD patients, achieving up to 80% correction and long-lasting effects after transplantation in mice. This approach improved blood health (even when only about 20% of the cells were corrected), avoided DNA damage seen in traditional CRISPR, and could offer a safe, one-time treatment for Sickle cell disease (SCD).^235^

## 5 Conclusion and future prospects

Genetic modification is quickly shaping our world—from better crops to medicines. The heart of this revolution is CRISPR-Cas9, a powerful tool making gene editing faster, easier, and more precise. For example, CRISPR is used in diagnosis, real-time tracking and visualization, and in advancing cancer immunotherapy. It can also help fortify crops by engineering genes that boost vitamin production, improve yield, and increase resilience to environmental stress, supporting global efforts to end hunger and improve nutrition by 2030.^236^ CRISPR tools are also helping to detect diseases like Zika,^44^ especially in remote areas, as outbreaks and reemergence of tropical diseases (TDs) often impact remote and rural communities with limited access to medical diagnostic tools. With support from e-healthcare and telemedicine, we can further boost access to such innovations. To use CRISPR safely and effectively, we need a comprehensive understanding of how its components work and how to deliver it where it's needed. CRISPR has enormous potential, but challenges like safe delivery, accuracy, precision, off-target effects and ethical concerns remain, and overcoming these is key to its safe, widespread use.

As technology evolves, its integration with emerging innovations, especially artificial intelligence and nanotechnology, could unlock personalized, powerful breakthroughs in medicine and beyond. Combining CRISPR biosensors with artificial intelligence (AI), cloud tech, the Internet of Things (IoT) and the Industrial Internet of things (IIoT) can make real-time health monitoring a reality.^237^ But to get there, we still need to improve things like accuracy, data privacy, and portability.^168^ In the future, simple, all-in-one diagnostic tools and smarter imaging could make CRISPR-based healthcare faster, easier, and more accessible for everyone. Future research may combine CRISPR with cell-based drug delivery combining gene editing with the natural ability of cells for personalized treatments. Scientists are also exploring its use to bring back extinct species, which could help restore ecosystems. With continued research, these innovations could provide a more sustainable future for both our planet and future generations.

## Author contributions

Nisha Bharti: literature review and writing – original draft preparation, Unnati Modi: literature review and writing – original draft preparation, Dhiraj Bhatia: conceptualization, writing – review and editing, and supervision. Raghu Solanki: conceptualization, writing – review and editing. All authors confirmed the final version of the manuscript.

## Conflicts of interest

The authors declare that they have no conflict of interest.

## Data Availability

No primary research results, software or code have been included and no new data were generated or analysed as part of this review.
